# Characterization of Microbial Degradation Products of Steviol Glycosides

**DOI:** 10.3390/molecules26226916

**Published:** 2021-11-16

**Authors:** Gert Steurs, Nico Moons, Luc Van Meervelt, Boudewijn Meesschaert, Wim Michel De Borggraeve

**Affiliations:** 1 Division of Molecular Design and Synthesis, Department of Chemistry, KU Leuven, Celestijnenlaan 200f, P.O. Box 2404, 3001 Leuven, Belgium; gert.steurs@kuleuven.be (G.S.); nico.moons1@gmail.com (N.M.); 2 Division of Biochemistry, Molecular and Structural Biology, Department of Chemistry, KU Leuven, Celestijnenlaan 200f, P.O. Box 2404, 3001 Leuven, Belgium; luc.vanmeervelt@kuleuven.be; 3 Laboratory for Microbial and Biochemical Technology, Department of Microbial and Molecular Systems, KU Leuven Bruges Campus, Spoorwegstraat 12, P.O. Box 7913, 8200 Brugge, Belgium; boudewijn.meesschaert@kuleuven.be

**Keywords:** steviol glycoside degradation, microbial degradation, soil bacteria, NMR, X-ray structure, GIAO NMR calculations, *monicanone*, *monicanol*

## Abstract

Steviol glycosides were subjected to bacteria present in a soil sample collected from a Stevia plantation in Paraguay. During the incubation experiments, next to the aglycon steviol, steviol degradation products were also formed. X-ray analysis and NMR methods in combination with chemical synthesis and GIAO NMR calculations were used to fully characterize the structure of these compounds as a tricyclic ketone and the corresponding reduced form. They were nicknamed *monicanone* and *monicanol*. The latter has the (*S*)-configuration at the alcohol site.

## 1. Introduction

Steviol glycosides (SGs) are sweet substances isolated from the leaves of the shrub *Stevia rebaudiana* (Bertoni) Bertoni. They can be used as non-caloric sweeteners since they do not degrade in the first part of the gastro-intestinal tract [1,2,3]. To elucidate possible fates of these compounds in nature, SGs were contacted with microorganisms extracted from a soil sample obtained from a Paraguayan Stevia plantation. The idea was that bacteria able to degrade SG would accumulate in soils that were repeatedly contacted with plant material containing these substances. In a three-step procedure, a bacterial consortium was isolated that converted a mixture of SGs containing mostly stevioside (60% *m/m*), rebaudioside A (28% *m/m*) and rebaudioside C (9% *m/m*) to a new and previously unknown compound [4]. At first, 5% *m/v* soil (99% dry matter) was incubated in 100 mL of steviol glycoside minimal growth medium (SMM) containing 0.02% *m/v* yeast extract and 0.1% *m/v* SGs. Within three days, the SGs mixture was completely converted into steviol. Prolonging the incubation time to one week resulted in full degradation of the previously formed steviol. Repeated sub-cultivation of the bacterial population that developed from the soil sample in SMM (10% inoculum) at the moment of full conversion of the SGs to steviol furnished a stable bacterial community capable of converting SGs nearly quantitatively to steviol in one week. This consortium was routinely used for the bio-organic synthesis of steviol. When the incubation with this consortium was extended to four weeks, a new compound was observed that was also detected in the prolonged incubation with the soil sample, indicating that the responsible micro-organism(s) was/were already present in the original soil sample. Repeated resuspension of the bacterial suspension (0.1 L) in 0.9 L of SMM at the moment of attaining the maximum concentration of this new compound (eight times) led to the development of a new bacterial consortium that converted the SGs from SMM nearly quantitively to this new compound in twelve days. This new bacterial consortium could be used for repetitive preparations of this new compound. From one of the first preparations, a second unknown compound could be isolated as well. Preliminary NMR and IR experiments allowed for the tentative structure assignment of these new compounds as a tricyclic ketone and its corresponding alcohol form, referred to as *monicanone* and *monicanol*, respectively. The isolated ketone was also shown to be present during the degradation of SGs by the microbial activity of UASB effluent (upstream anaerobic sludge blanket reactor). This may indicate that the ketone is also an intermediate during the environmental cleanup of SGs [4]. The isolation and identification of these two new compounds has already been reported in our previous work. Full structural assignment, however, was not established, as this was beyond the scope of the article.

In this paper, we confirm the initially assigned structure of these compounds and assign the absolute stereochemistry of the reduced form using a combination of GIAO NMR calculations and organic synthesis.

## 2. Results and Discussion

### 2.1. Preparation

**Scheme 1:**
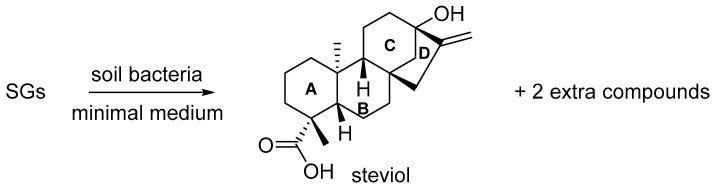
Incubation of steviol glycosides (consisting mostly of stevioside (60% *m/m*), rebaudioside A (28% *m/m*) and rebaudioside C (9% *m/m*)) with soil bacteria gave rise to formation of steviol (with indication of ring nomenclature) and two extra degradation compounds [4].

The preparation and isolation of the two new degradation compounds via microbial degradation (Scheme 1) is described in recent work by some of the coauthors of this paper [4]. On TLC, the first compound has an *R*_f_ value of 0.40 in a 50/50 mixture of *n*-hexane/ethyl acetate, while the second compound has an *R*_f_ of 0.21 in the same solvent system. The compounds can be visualized using Hanessian’s staining agent.

### 2.2. Characterization of the First Compound: Monicanone

Comparison of the ^13^C-NMR data of the first compound isolated (Table 1) with the NMR data of steviol [5] showed that the signals of the A ring were missing. Moreover, a clear new signal was present at 212.89 ppm, indicative for a ketone (hence the compound was nicknamed *monicanone*). This was confirmed in the infrared spectrum where a peak was observed at 1687 cm^−1^. High resolution mass spectrometry revealed a *m*/*z* ratio of 220.1463, which is consistent with C_14_H_20_O_2_ [4]_._

#### 2.2.1. NMR Analysis

**Figure 1:**
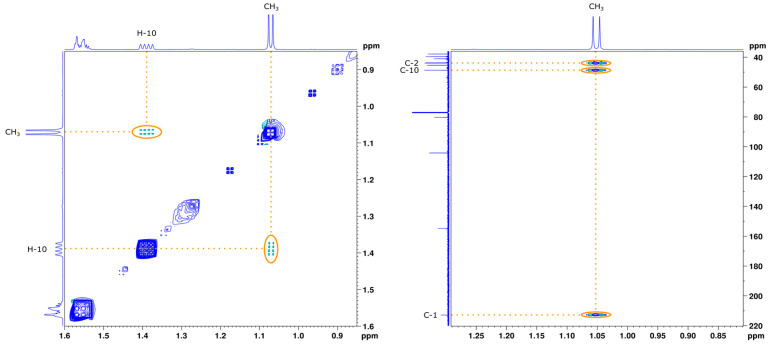
Zoom of the NOESY (left) and HMBC (right) spectrum of monicanone in CDCl_3_. Full spectra are available in Supplementary Materials S1 pages S-8–S-9 .

One-dimensional NMR data of monicanone are summarized in Table 1. Additional 2D NMR spectra (see also Supplementary Materials S1 pages S-4–S-9 https://www.mdpi.com/article/10.3390/molecules26226922/s1) allowed us to propose the full structure shown in Table 1. The presence of an NOE effect between the 14-Me group and proton 10 (Figure 1) is indicative for the inverted stereochemistry of this methyl group with respect to the parent steviol core. The large 12.2 Hz coupling constant between H-1 and H-10 further confirms a trans diaxial disposition of these two protons on the ring. In the HMBC spectrum, the protons of the methyl group showed interaction via ^2^*J*_CH_ with C-1 and via ^3^*J*_CH_ with C-2 and C-10. The methyl (H-14) to C-2 cross peak also allowed us to determine the position of the ketone in the structure.

#### 2.2.2. X-ray Analysis

**Figure 2:**
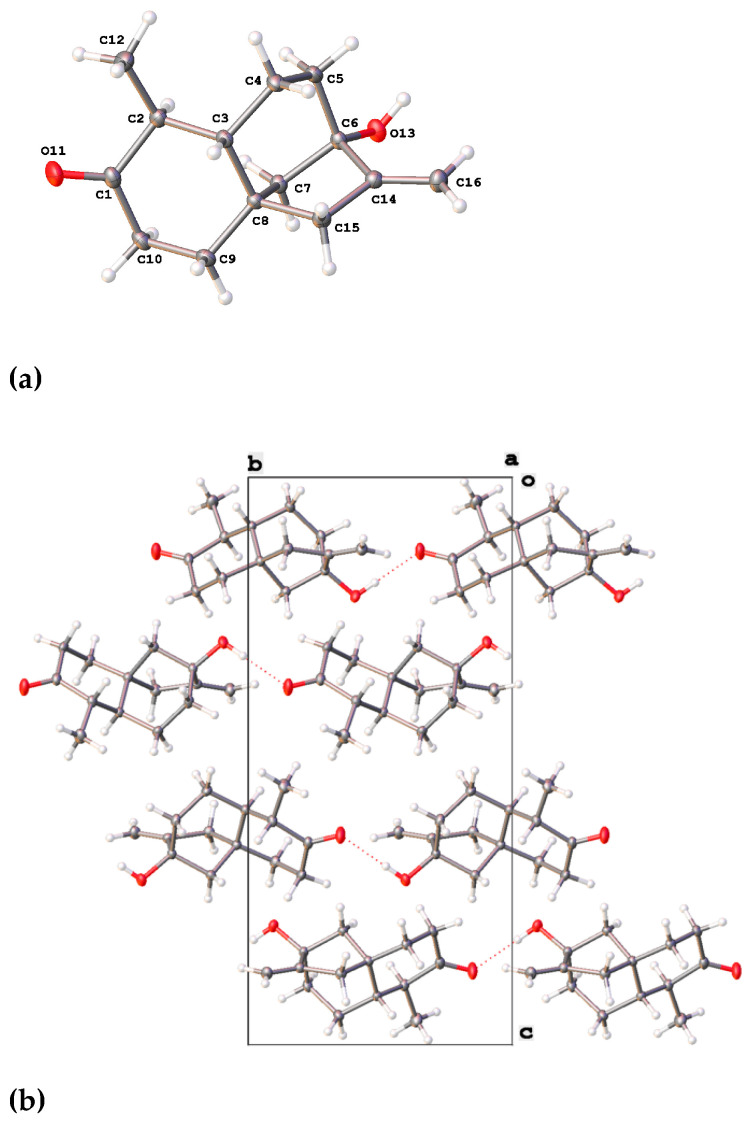
(**a**) X-ray structure of monicanone with ellipsoids drawn at the 50% probability level. (**b**) A view down the *a* axis of the crystal packing showing the O-H∙∙∙O interactions (red dashed lines).

The NMR-derived structure was later confirmed after we succeeded in obtaining a crystal of monicanone suitable for X-ray diffraction via slow evaporation of a concentrated methanol solution of monicanone open to the air. The X-ray structure is shown in Figure 2. The crystal packing is stabilized by O-H∙∙∙O interactions between the keto and alcohol functions (O13-H13: 0.84(3) Å, H13∙∙∙O11: 1.99(3) Å, O13∙∙∙O11: 2.827(3) Å, O13-H13∙∙∙O11: 176(3)°), resulting in chains of molecules running in the *b* direction. The five-membered ring (C6-C8, C14-C15) has an envelope conformation with C7 as the flap (puckering parameters Q(2) = 0.472(2) Å, φ(2) = 216.5(3)°). The puckering parameters for the six-membered rings (for ring C1-C3, C8-C10: Q = 0.538(2) Å, θ = 10.1(2)°, φ = 195.1(14)° and for ring C3-C8: Q = 0.645(2) Å, θ = 161.80(18)°, φ = 57.5(6)°) illustrate the chair conformation of both rings. Substituents O11, C12, O13 and C16 are in equatorial positions.

### 2.3. Characterization of the Second Compound: Monicanol

#### 2.3.1. NMR Analysis

**Figure 3:**
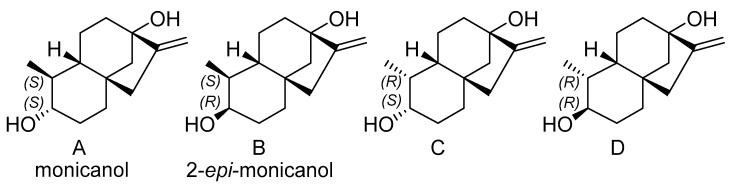
Proposed structures for the second compound isolated from the fermentation broth.

**Figure 4:**
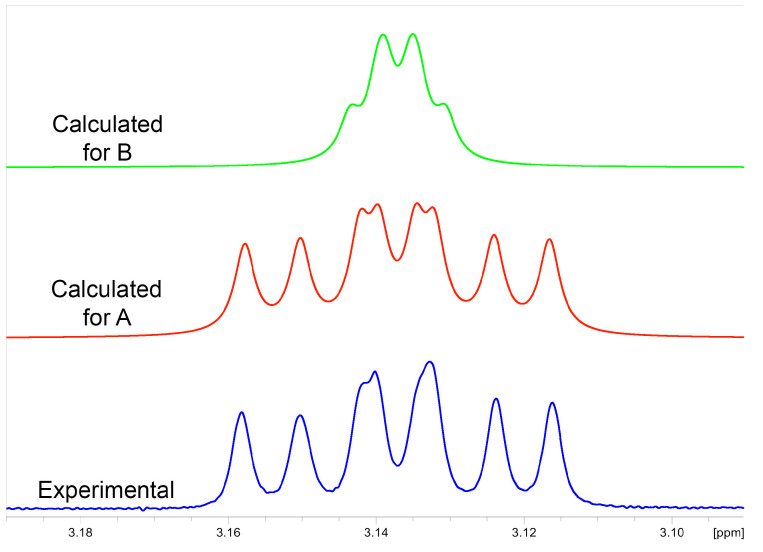
Comparison of the experimental ^1^H NMR multiplet at 3.13 ppm of monicanol (blue) with simulated multiplet patterns using scaled calculated coupling constants for diastereomer A (red, scaled coupling constants 10.90, 9.34 and 4.52 Hz, monicanol) and compound B (green, scaled coupling constants 3.00, 2.16 and 2.63 Hz, 2-*epi*-monicanol).

According to ^13^C-NMR analysis, the second compound with a nominal molecular mass of 222 Da was structurally related to monicanone. Striking differences, however, were the lack of the ketone functionality and the appearance of a new signal at 76.57 ppm. This in combination with a new signal at 3.13 ppm in the ^1^H NMR spectrum was highly indicative of the structure of the corresponding alcohol of monicanone (which was later nicknamed *monicanol*). Assuming that this alcohol is a precursor for monicanone, we hypothesized that it could be one of four diastereomers depicted in Figure 3 (we were also not sure at what stage methyl-epimerization occurs in the degradation pathway starting from the SGs). GIAO ^13^C-NMR calculations in chloroform were run (see experimental section) and the calculated chemical shifts for each diastereomer were compared with experimental data simply by numerically ordering the chemical shifts and making the sum of absolute values of pairwise differences between calculated and experimental shifts (Table 2). Diastereomer A best agreed with the experimental NMR data. Moreover, the calculated proton chemical shift value of 3.09 ppm for the C*H*OH (proton H-2) of this compound was in remarkable agreement with the experimental value of 3.19 ppm. Finally, in this proposed structure, the C*H*OH was expected to show two large ^3^*J*_ax,ax_ and one small ^3^*J*_ax,eq_ with the neighboring protons, which is confirmed in the experimental spectrum (coupling to OH is not observed due to exchange). Simulation of this multiplet shape with values calculated for the coupling constants using a procedure described by Bally et al. [6] also shows a remarkable agreement with the experiment for compound **A** (Figure 4).

**Figure 5:**
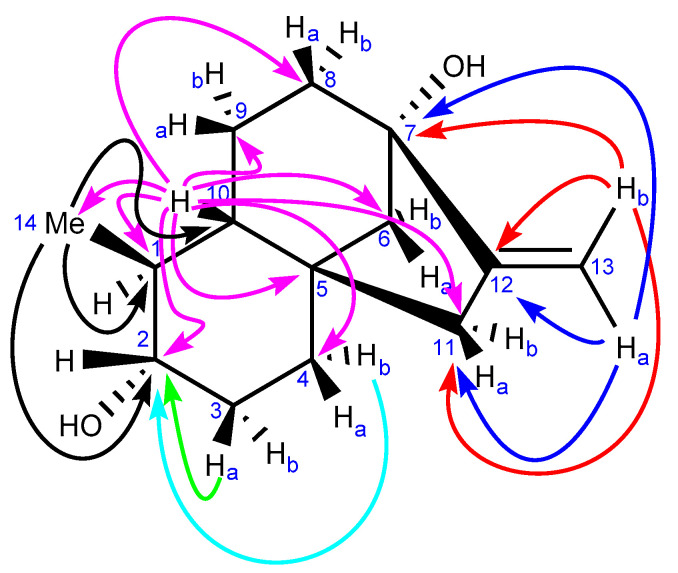
Key correlations in the HMBC spectrum of the second new compound isolated from the broth (monicanol).

Further 2D NMR experiments (e.g., correlations from HMBC spectra as shown in Figure 5 and spectra in Supplementary Materials S1 pages S-10–S-14 https://www.mdpi.com/article/10.3390/molecules26226922/s1) are also in complete agreement with the proposed structure A for monicanol and allowed us to assign all signals according to the data in Table 3.

#### 2.3.2. Chemical Synthesis of Monicanol and 2-*epi*-Monicanol

**Figure 6:**
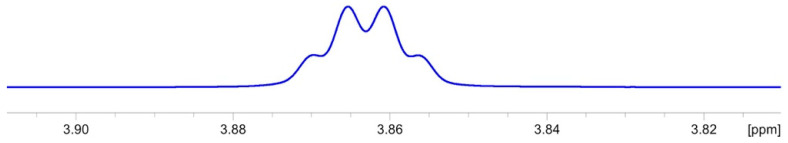
Experimental ^1^H NMR multiplet pattern of H-2 of 2-*epi*-monicanol.

After obtaining an extra batch of monicanone, we were able to chemically reduce its ketone with NaBH_4_ (*vide infra*, Experimental Section) to form a mixture of the corresponding diastereomeric alcohols (ratio 72:28). The major one of these compounds was indeed identical to the second compound isolated from the fermentation broth, confirming our initial structure suggestion. As expected, H-2 in 2-*epi*-monicanol displays a narrower multiplet in ^1^H NMR (Figure 6) because of the absence of large ^3^*J*_ax,ax_ coupling constants (Table 3).

This allowed us to conclude that the alcohol compound isolated initially as a contaminant from the main component monicanone was diastereomer A and had the (*S*)-configuration at the new alcohol site (Figure 3). The 2-OH epimer (2-*epi*-monicanol) was never isolated from the broth. However, we cannot fully exclude that it was never present in the broth during the fermentation process.

## 3. Experimental Section

### 3.1. Reagents and Materials

All reagents and solvents were purchased from Fisher Scientific or Acros Organics and were used without any further purification. All reactions were performed under air atmosphere and stirred magnetically with PTFE-coated magnetic stirring bars at 350–450 rpm. Solvents were evaporated under reduced pressure using a rotary evaporator at a bath temperature of 50 °C. Final compounds were dried under high vacuum (10^−3^ mbar) at room temperature. Yields refer to isolated compounds after purification.

Samples of the degradation compounds were provided by our collaborators and obtained as described in Reference [4] (see Introduction).

### 3.2. Methods

#### 3.2.1. Thin-Layer Chromatography

Thin-layer chromatography (TLC) analysis was performed using Sigma-Aldrich 20 × 20 cm precoated, glass TLC plates with fluorescent indicator at 254 nm (article number 99571: layer thickness 250 μm, particle size 8.0–12.0 μm, average pore diameter 60 Å). Visualization of monicanone and monicanol was performed using Hanessian’s staining agent.

#### 3.2.2. Column Chromatography

Column chromatography was performed using Acros Organics silica gel for chromatography article number 240370300, particle size 0.060–0.200 mm, average pore diameter 60 Å).

#### 3.2.3. Preparative High-Performance Liquid Chromatography

Preparative high-performance liquid chromatography (prep-HPLC) was performed using an Agilent 1100 HPLC system, consisting of a G1311A quaternary pump and solvent module and a G1315A diode-array detector (DAD, operating at 215 nm). The HPLC system was equipped with a Phenomenex Luna reversed-phase C18 column (particle size 5 µm, pore size 100 Å, length 150 mm, ID 21.20 mm). Data were acquired using Agilent LC/MSD ChemStation software rev. B.04.03 and processed and analyzed using ACD/Spectrus Processor 2019.1.2.

#### 3.2.4. Nuclear Magnetic Resonance Spectroscopy

Nuclear magnetic resonance (NMR) spectra were recorded on a Bruker Avance II^+^ 600 spectrometer with a Bruker 600 UltraShield^TM^ magnet system (^1^H basic frequency of 600.13 MHz). ^1^H-detected data were acquired using a 5 mm PATXI 600S3 H-C/N-D probe with z-gradients, while ^13^C-detected data were acquired using a 5 mm PABBO BB (31P-109Ag)-1H/D probe with z-gradients. ^13^C-detected experiments were ^1^H-decoupled using power-gated broadband decoupling. All samples were dissolved in chloroform-*d* (CDCl_3_). Data were recorded at 298 K using Bruker TopSpin 3.6.1 and processed and analyzed using Bruker TopSpin 4.1.1. All spectra were calibrated using tetramethylsilane (TMS) as an internal reference (0.00 ppm for ^1^H and ^13^C). The δ-values are expressed in parts per million (ppm). The following acronyms (alone or in combination) were used: d (doublet), t (triplet), q (quartet), m (multiplet).

#### 3.2.5. Infrared Spectroscopy

Attenuated total reflection (ATR) Fourier-transformed infrared (FT-IR) spectra were recorded on a Bruker Alpha-P FT-IR spectrometer with single reflection Platinum ATR accessory. Samples were analyzed neat in solid or liquid state without any further manipulations. The data were recorded at room temperature using Bruker OPUS 7.5 and processed and analyzed using ACD/Spectrus Processor 2019.1.2. The ν-values are reported in units of reciprocal centimeters (cm^−1^).

#### 3.2.6. Ultraviolet/Visible Light Spectroscopy

Ultra-violet/visible light (UV/Vis) absorption spectra reported in Supplementary Materials S1 pages S-20–S-21 (https://www.mdpi.com/article/10.3390/molecules26226922/s1) were recorded on a Varian Cary 5000 UV-Vis-NIR spectrophotometer in double-beam mode. All measurements were performed at 20 °C in quartz cuvettes with an optical path length of 1 mm. Data were collected using Varian’s WinUV software’s Scan package version 3.00 and processed using GraphPad Prism 9.2.0. Wavelengths are expressed in nanometers (nm), while molar extinction coefficients are expressed in L mol^−1^ cm^−1^.

#### 3.2.7. Circular Dichroism Spectroscopy

Circular dichroism (CD) spectra reported in Supplementary Materials S1 pages S-22–S-23 (https://www.mdpi.com/article/10.3390/molecules26226922/s1) were recorded on a JASCO J-1500 circular dichroism spectrophotometer. All measurements were performed at 20 °C in quartz cuvettes with an optical path length of 1 mm. Data were collected using JASCO’s Spectra Manager software version 2.12.00 and processed using GraphPad Prism 9.2.0.

#### 3.2.8. High-Resolution Mass Spectrometry

High-resolution mass spectra (HR-MS) reported in Supplementary Materials S1 page S-2 (https://www.mdpi.com/article/10.3390/molecules26226922/s1) were acquired on a Waters Synapt G2 HDMS quadrupole orthogonal-acceleration time-of-flight (Q-oa-ToF) mass spectrometer with an electrospray ionization (ESI) source (capillary voltage 4000 V), operating in the positive mode. Data were recorded with a resolution of 15,000 (FWHM) using leucine encephalin as lock mass. Samples were prepared by dissolving the compound in methanol and diluting this solution with an acetonitrile/water mixture (1/1 *v/v*) to an approximate concentration of 0.1 mM. Each sample was infused at a flow rate of 5 µL/min. Data were acquired and processed using Waters MassLynx^TM^ 4.1 software.

#### 3.2.9. Low-Resolution Mass Spectrometry

Low-resolution mass spectra (LR-MS) reported in Supplementary Materials S1 page S-3 (https://www.mdpi.com/article/10.3390/molecules26226922/s1) were recorded using an Agilent 1100 HPLC system, consisting of a G1311A quaternary pump and solvent module, a G1313A automatic liquid sampler (ALS), a G1315A diode-array detector (DAD, operating at 215, 254, 280, 320 and 365 nm) and a G1316A thermostated column compartment (TCC, kept at a constant temperature of 25 °C) without an HPLC column (direct injection method). The HPLC system was coupled to an Agilent 6110 single-quadrupole mass spectrometer with an electrospray ionization (ESI) source (capillary voltage 3500 V), operating in the positive mode. Samples were prepared by dissolving the compound in methanol to an approximate concentration of 1 mM. Each sample was automatically injected onto the HPLC system (injection volume 10 µL) and run isocratically in 100% methanol (LC-MS grade, Fisher Scientific) with a flow rate of 0.2 mL/min. Data were acquired using Agilent LC/MSD ChemStation software rev. B.04.03-SP2 and processed and analyzed using ACD/Spectrus Processor 2019.1.2.

#### 3.2.10. Single-Crystal X-ray Crystallography

A single crystal of monicanone was obtained by slowly evaporating a solution of monicanone in methanol. In the solids formed, a crystal of sufficient quality was found to run the X-ray diffraction experiment.

X-ray intensity data were collected at 293(2) K on an Agilent SuperNova diffractometer with Eos CCD detector using MoKα radiation. The images were processed (unit cell determination, intensity data integration, correction for Lorentz and polarization effects, and empirical absorption correction) using CrysAlisPRO [7]. The structure was solved using Olex2 [8] with the ShelXT [9] structure solution program using Intrinsic Phasing and refined with the ShelXL [10] refinement package using full-matrix least-squares minimization on F^2^. Non-hydrogen atoms were refined anisotropically. Hydrogen atoms were located from difference Fourier maps and refined with isotropic temperature factors. The value of the Flack parameter of 0.4(4) did not allow us to reliably determine the absolute stereochemistry due to a lack of suitable anomalous scatterers. However, the absolute configuration could be assigned by comparison with the known stereochemistry of the starting materials. Crystallographic data were deposited in the Cambridge Crystallographic Data Centre (CCDC) with deposition number CCDC 2111608. These can be obtained free of charge from the CCDC via https://www.ccdc.cam.ac.uk/structures (accessed on 9 November 2021).

#### 3.2.11. NMR Calculations

GIAO NMR calculations were performed using Gaussian 16 Rev A.03 [11]: the chemical shifts were calculated following a two-step procedure [12]. First, a geometry optimization of the proposed structures was performed using DFT calculations with a B3LYP/6-31+G(d,p) basis set in the gas phase. Via a frequency calculation, the system was checked for the absence of imaginary frequencies to ensure a local minimum was reached. Shielding values were then calculated for the optimized geometry using the GIAO method with the mPW1PW91/6-311+G(2d,p) basis set using the SMD variation of the IEFPCM solvent model for chloroform. The calculated isotropic shielding values were finally converted to chemical shifts using scaling factors taken from https://cheshirenmr.info (accessed on 9 November 2021) according to Equations (1) and (2).

^1^H NMR: (scaled chemical shift) = (31.8018 − (unscaled shielding))/1.0936 (1)

^13^C NMR: (scaled chemical shift) = (186.5242 − (unscaled shielding))/1.0533 (2)

Multiplet structures were simulated using the Bruker TopSpin 4.1.3 software using the DAISY package with scaled coupling constants obtained from the NMR calculations.

#### 3.2.12. Reduction of Monicanone—Synthesis of Monicanol and 2-*epi*-Monicanol

In a round-bottom flask, monicanone (55.1 mg, 0.25 mmol, 1.00 equiv.) was dissolved in EtOH (4.0 mL). To this solution, NaBH_4_ (37.8 mg, 1.00 mmol, 4.00 equiv.) was added while stirring at ambient temperature. The solution was stirred for 3 h and subsequently quenched by the addition of 10 mL of water. The resulting mixture was extracted 3 x with DCM. The organic layers were combined, washed with brine, dried over anhydrous MgSO_4_ and filtered. The filtrate was concentrated under reduced pressure. ^1^H NMR analysis of the crude residue showed the presence of monicanol and 2-*epi*-monicanol in a ratio of 72:28. Isolation of both diastereomers was performed using preparative HPLC. Therefore, the crude residue was dissolved in 1 mL of HPLC-grade MeOH and loaded onto the HPLC system. The sample was run in H_2_O + 0.1% HCOOH/MeOH at a flow rate of 5.00 mL/min (0–30 min linear gradient from 50% MeOH to 100% MeOH, then 10 min isocratically 100% MeOH; UV detection at 215 nm), yielding 18.0 mg of pure monicanol and 4.2 mg of a mixture of mainly 2-*epi*-monicanol with a small portion of monicanol (ratio 88:12). Both diastereomers were obtained as white solids in a combined isolated yield of 40%.

## Figures and Tables

**Table 1:**
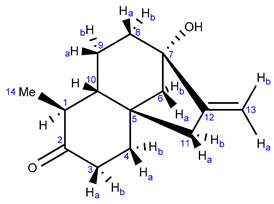
^1^H (600 MHz) and ^13^C (151 MHz) NMR data and structure of monicanone. Spectra are recorded at 298 K in CDCl_3_. Coupling constants are matched amongst the different multiplets. For the unmatched coupling constants, please refer to Supplementary Materials S1 page S-2. First-order analysis has been applied.

Position	Monicanone	
	$\delta_{1_{H}}$	$\delta_{13_{C}}$
**1**	2.51 (dq, *J* = 12.3, 6.5 Hz)	43.91
**2**		212.89
**3**	a: 2.40 (m) b: 2.44 (tdd, *J* = 13.8, 6.1, 1.0 Hz)	39.61
**4**	a: 1.86 (td, *J* = 13.8, 5.4 Hz) b: 1.72 (m)	37.83
**5**		41.05
**6**	a: 1.54 (m) b: 2.21 (dd, *J* = 10.6, 2.5 Hz)	45.37
**7**		80.31
**8**	a: 1.53 (m) b: 1.81 (m)	35.72
**9**	a: 1.71 (m) b: 1.78 (m)	23.06
**10**	1.37 (dd, *J* = 12.3, 6.1 Hz)	48.68
**11**	a: 2.25 (ddd, *J* = 17.0, 2.5, 2.1 Hz) b: 2.36 (dt, *J* = 17.0, 2.7 Hz)	43.74
**12**		154.72
**13**	a: 4.91 (t, *J* = 2.1 Hz) b: 5.07 (t, *J* = 2.7 Hz)	104.17
**14**	1.05 (d, *J* = 6.5 Hz)	11.92

**Table 2:** Comparison of experimental chemical shifts for the second isolated compound and GIAO ^13^C-NMR chemical shifts calculated for proposed structures A–D (See also Supplementary Materials S2 for calculation details).

Isolated	Diastereomer A		Diastereomer B		Diastereomer C		Diastereomer D	
$\delta_{13_{C,exp}}$	$\delta_{13_{C,A}}$	$\left|\delta_{13_{C,exp}}-\delta_{13_{C,A}}\right|$	$\delta_{13_{C,B}}$	$\left|\delta_{13_{C,exp}}-\delta_{13_{C,B}}\right|$	$\delta_{13_{C,C}}$	$\left|\delta_{13_{C,exp}}-\delta_{13_{C,C}}\right|$	$\delta_{13_{C,D}}$	$\left|\delta_{13_{C,exp}}-\delta_{13_{C,D}}\right|$
15.63	14.14	1.49	15.77	0.14	9.39	6.24	15.56	0.07
21.88	22.98	1.10	22.88	1.00	26.60	4.72	25.89	4.01
32.63	33.82	1.19	32.24	0.39	29.29	3.34	28.02	4.61
36.18	35.62	0.56	33.04	3.14	36.44	0.26	31.72	4.46
36.45	38.27	1.82	36.06	0.39	41.50	5.05	38.28	1.83
38.26	38.91	0.65	38.96	0.70	42.07	3.81	41.63	3.37
41.03	43.96	2.93	41.26	0.23	43.29	2.26	42.17	1.14
44.56	44.66	0.10	44.00	0.56	43.96	0.60	43.38	1.18
45.90	45.35	0.55	44.93	0.97	46.06	0.16	46.52	0.62
46.27	46.97	0.70	45.13	1.14	47.03	0.76	46.85	0.58
76.57	74.93	1.64	72.31	4.26	73.13	3.44	72.75	3.82
80.48	80.85	0.37	80.95	0.47	80.81	0.33	80.89	0.41
103.44	102.41	1.03	102.43	1.01	102.47	0.97	102.02	1.42
155.72	158.36	2.64	158.75	3.03	158.18	2.46	158.40	2.68
**Sum of differences**		**16.77**		**17.43**		**34.40**		**30.20**

**Table 3:**
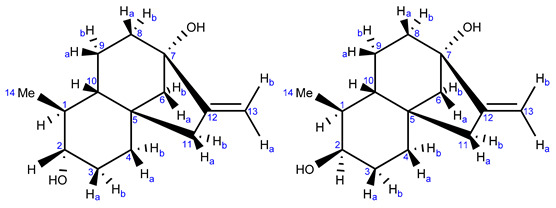
^1^H (600 MHz) and ^13^C (151 MHz) NMR data for monicanol and 2-*epi*-monicanol. Spectra are recorded at 298 K in CDCl_3_. Coupling constants are matched amongst the different multiplets. For the unmatched coupling constants, please refer to Supplementary Materials S1 pages S-2–S-3. First-order analysis has been applied.

Position	Monicanol		2-*epi*-monicanol	
	$\delta_{1_{H}}$	$\delta_{13_{C}}$	$\delta_{1_{H}}$	$\delta_{13_{C}}$
**1**	1.54 (m)	38.26	1.77 (dqd, *J* = 12.1, 6.8, 2.8 Hz)	34.16
**2**	3.13 (ddd, *J* = 10.9, 9.7, 4.7 Hz)	76.57	3.84 (dd, *J* = 5.8, 2.8 Hz)	71.88
**3**	a: 1.87 (m) b: 1.47 (m)	32.63	a: 1.76 (ddd, *J* = 14.2, 5.8, 3.8 Hz) b: 1.66 (tdd, *J* = 14.2, 4.3, 2.7 Hz)	30.80
**4**	a: 1.55 (td, *J* = 12.8, 2.9 Hz) b: 1.41 (dt, *J* = 12.8, 3.2 Hz)	36.18	a: 1.87 (ddd, *J* = 14.2, 13.2, 3.8 Hz) b: 1.15 (ddd, *J* = 13.2, 4.3, 2.7 Hz)	32.16
**5**		41.03		41.26
**6**	a: 1.30 (ddd, *J* = 10.8, 3.2, 1.2 Hz) b: 2.00 (dd, *J* = 10.8, 2.7 Hz)	45.90	a: 1.27 (ddd, *J* = 10.8, 3.1, 1.1 Hz) b: 1.98 (dd, *J* = 10.8, 2.3 Hz)	45.34
**7**		80.48		80.56
**8**	a: 1.44 (dddd, *J* = 11.3, 5.3, 3.2, 1.7 Hz) b: 1.69 (ddd, *J* = 13.7, 11.3, 4.9 Hz)	36.45	a: 1.44 (m) b: 1.67 (m)	36.38
**9**	a: 1.59 (m) b: 1.80 (ddt, *J* = 14.2, 4.9, 1.7 Hz)	21.88	a: 1.62 (m) b: 1.68 (m)	21.64
**10**	0.98 (dd, *J* = 11.5, 6.5 Hz)	46.27	1.45 (dd, *J* = 12.1, 6.1 Hz	40.57
**11**	a: 2.17 (ddd, *J* = 17.0, 4.6, 2.3 Hz) b: 2.24 (dt, *J* = 17.0, 2.7 Hz)	44.56	a: 2.25 (ddd, *J* = 17.1, 4.5, 2.3 Hz) b: 2.21 (dt, *J* = 17.1, 2.7 Hz)	44.95
**12**		155.72		156.00
**13**	a: 4.85 (ddt, *J* = 2.6, 1.9, 0.7 Hz) b: 5.00 (ddd, *J* = 3.0, 2.3, 0.7 Hz)	103.44	a: 4.85 (ddt, *J* = 2.7, 2.3, 0.7 Hz b: 4.98 (ddd, *J* = 2.7, 2.3, 0.7 Hz)	103.25
**14**	1.03 (d, *J* = 6.3 Hz)	15.63	0.99 (d, *J* = 6.8 Hz)	16.43

## Data Availability

Not applicable.

## Supplementary Materials

The following are available online at https://www.mdpi.com/article/10.3390/molecules26226922/s1, characterization data of the compounds (Supplementary_Materials_S1_Characterization_of_microbial_degradation_products_of_steviol_glycosides.pdf), input and output files of the NMR calculations (Supplementary_Materials_S2_GIAO_NMR_Calculations.zip).
