# Supplementary material for: Characterization of Microbial Degradation Products of Steviol Glycosides
Source: Molecules. 2021 Nov 16;26(22):6916. doi: 10.3390/molecules26226916 (PMC8625998; doi:10.3390/molecules26226916)
Supplement: Supplementary file 1 [file molecules-26-06916-s001.zip › molecules-1427844-supplementary/Supplementary_Materials_1_Characterization_of_microbial_degradation_products_of_steviol_glycosides.pdf]

Characterization of microbial degradation products of steviol glycosides

Gert Steurs [1], Nico Moons [1], Luc Van Meervelt [2], Boudewijn Meesschaert [3], and Wim Michel De Borggraeve [1,*]

[1] Department of Chemistry, Division of Molecular Design and Synthesis, KU Leuven, Celestijnenlaan 200f box 2404, 3001 Leuven, Belgium; gert.steurs@kuleuven.be (G.S.); nico.moons1@gmail.com (N.M.)

[2] Department of Chemistry, Division of Biochemistry, Molecular and Structural Biology, KU Leuven, Celestijnenlaan 200f box 2404, 3001 Leuven, Belgium; luc.vanmeervelt@kuleuven.be

[3] Department of Microbial and Molecular Systems, Laboratory for Microbial and Biochemical Technology, KU Leuven Bruges Campus, Spoorwegstraat 12 box 7913, 8200 Brugge, Belgium; boudewijn.meesschaert@kuleuven.be

* Correspondence: wim.deborggraeve@kuleuven.be

Supplementary Materials

1. Content

1. Content S-1
2. Summary of the characterization of the compounds S-2
   2.1. Monicanone S-2
   2.2. Monicanol S-2
   2.3. 2-*epi*-monicanol S-3
3. NMR spectra of Monicanone, Monicanol and 2-*epi*-Monicanol S-4
   3.1. Monicanone S-4
   3.2. Monicanol S-10
   3.3. 2-*epi*-monicanol S-15
4. UV/Vis spectra of monicanone, monicanol and 2-*epi*-monicanol S-20
   4.1. Monicanone S-20
   4.2. Monicanol S-20
   4.3. 2-*epi*-monicanol S-21
5. CD spectra of monicanone, monicanol and 2-*epi*-monicanol S-22
   5.1. Monicanone S-22
   5.2. Monicanol S-22
   5.3. 2-*epi*-monicanol S-23
6. XRD data of monicanone S-24

2. Summary of the characterization of the compounds

2.1. Monicanone

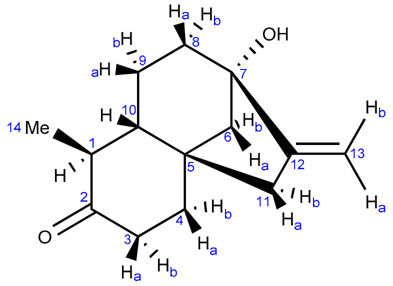

^{1}H NMR (600 MHz, $CDCl_3$) δ 5.07 (t, *J* = 2.6 Hz, 1H, H-13b), 4.91 (t, *J* = 2.2 Hz, 1H, H-13a), 2.51 (dq, *J* = 12.2, 6.4 Hz, 1H, H-2), 2.44 (tdd, *J* = 14.0, 6.1, 1.0 Hz, 1H, H-6b), 2.42-2.38 (m, 1H, H-6a), 2.36 (dt, *J* = 17.0, 2.7 Hz, 1H, H-12b), 2.25 (ddd, *J* = 17.0, 2.3, 2.0 Hz, 1H, H-12a), 2.21 (dd, *J* = 10.6, 2.6 Hz, 1H, H-10b), 1.86 (td, *J* = 13.5, 5.4 Hz, 1H, H-5a), 1.82-1.66 (m, 4H, H-8b (1.81), H-7b (1.78), H-5b (1.72), H-7a (1.71)), 1.56-1.51 (m, 2H, H-10a (1.54), H-8a (1.53)), 1.37 (dd, *J* = 12.4, 6.1 Hz, 1H, H-3), 1.05 (d, *J* = 6.5 Hz, 3H, H-14). **^{13}C NMR** (151 MHz, $CDCl_3$) δ 212.89 (C-1), 154.72 (C-11), 104.17 (C-13), 80.31 (C-9), 48.68 (C-3), 45.37 (C-10), 43.91 (C-2), 43.74 (C-12), 41.05 (C-4), 39.61 (C-6), 37.83 (C-5), 35.72 (C-8), 23.06 (C-7), 11.92 (C-14). **IR** (neat): ν 3350.51 (O-H stretch), 2924.72 (C-H stretch), 2854.45 (C-H stretch), 1686.62 (C=O stretch), 1659.17 (C=C stretch). **UV/Vis** ($CDCl_3$): λ_{max} (ε) 241 (87), 290 (42). **HR-MS** (ESI): m/z calculated for $[M+H]^+$ 221.1536, found 221.1514. **R_f** (*n*-hexane/EtOAc 5/5): 0.396. **Physical appearance**: white solid.

2.2. Monicanol

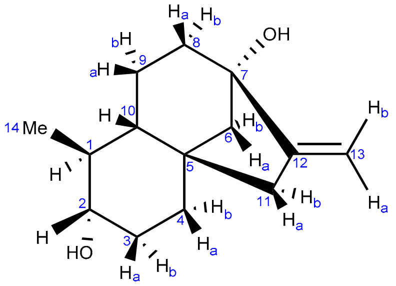

^{1}H NMR (600 MHz, $CDCl_3$): δ 5.00 (ddd, *J* = 3.0, 2.3, 0.7 Hz, 1H, H-13b), 4.85 (ddt, *J* = 2.6, 1.9, 0.7 Hz, 1H, H-13a), 3.13 (ddd, *J* = 10.9, 9.7, 4.7 Hz, 1H, H-1), 2.24 (dt, *J* = 17.0, 2.7 Hz, 1H, H-12b), 2.17 (ddd, *J* = 17.0, 4.6, 2.3 Hz, 1H, H-12a), 2.00 (dd, *J* = 10.8, 2.7 Hz, 1H, H-10b), 1.89-1.85 (m, 1H, H-6a), 1.80 (ddt, *J* = 14.2, 4.9, 1.3 Hz, 1H, H-7b), 1.69 (ddd, *J* = 13.7, 11.3, 4.9 Hz, 1H, H-8b), 1.63-1.55 (m, 1H, H-7a), 1.55 (td, *J* = 13.1, 2.9 Hz, 1H, H-5a), 1.58-1.50 (m, 1H, H-2), 1.51-1.43 (m, 1H, H-6b), 1.44 (dddd, *J* = 11.2, 5.3, 3.2, 2.0 Hz, 1H, H-8a), 1.41 (dt, *J* = 12.5, 3.2 Hz, 1H, H-5b), 1.30 (ddd, *J* = 10.8, 3.1, 1.2 Hz, 1H, H-10a), 1.03 (d, *J* = 6.3 Hz, 3H, H-14), 0.98 (dd, *J* = 11.5, 6.5 Hz, 1H, H-3). **^{13}C NMR** (151 MHz, $CDCl_3$): δ 155.72 (C-11), 103.44 (C-13), 80.48 (C-9), 76.57 (C-1), 46.27 (C-3), 45.90 (C-10), 44.56 (C-12), 41.03 (C-4), 38.26 (C-2), 36.45 (C-8), 36.18 (C-5), 32.63 (C-6), 21.88 (C-7), 15.63 (C-14). **IR** (neat): ν 3400.12 (O-H stretch), 3344.31 (O-H stretch), 2961.93 (C-H stretch), 2918.52 (C-H stretch), 2893.72 (C-H stretch), 2858.58 (C-H stretch), 1659.75 (C=C stretch). **HR-MS** (ESI): m/z calculated for $[M+H]^+$ 223.1692, found 223.1697. **R_f** (*n*-hexane/EtOAc 5/5): 0.208. **Physical appearance**: white solid.

2.3. 2-epi-monicanol

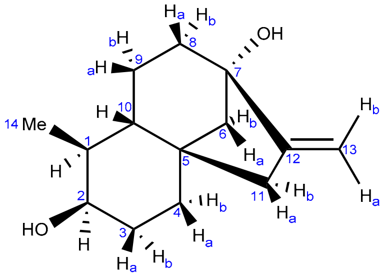

^{1}H NMR (600 MHz, $CDCl_3$): δ 4.98 (ddd, *J* = 3.0, 2.3, 0.7 Hz, 1H, H-13b), 4.85 (ddt, *J* = 2.5, 1.9, 0.7 Hz, 1H, H-13a), 3.84 (dd, *J* = 5.6, 2.8 Hz, 1H, H-1), 2.25 (ddd, *J* = 17.1, 4.5, 2.3 Hz, 1H, H-12a), 2.21 (dt, *J* = 17.1, 2.7 Hz, 1H, H-12b), 1.98 (dd, *J* = 10.8, 2.3 Hz, 1H, H-10b), 1.87 (ddd, *J* = 14.2, 13.2, 4.1 Hz, 1H, H-5a), 1.77 (dqd, *J* = 12.1, 6.8, 2.7 Hz, 1H, H-2), 1.76 (ddd, *J* = 14.0, 5.9, 3.4 Hz, 1H, H-6a), 1.70-1.66 (m, 1H, H-7b), 1.69-1.65 (m, 1H, H-8b), 1.66 (tdd, *J* = 14.2, 4.3, 2.7 Hz, 1H, H-6b), 1.64-1.59 (m, 1H, H-7a), 1.45 (dd, *J* = 12.1, 6.1 Hz, 1H, H-3), 1.46-1.42 (m, 1H, H-8a), 1.27 (ddd, *J* = 10.8, 3.1, 1.1 Hz, 1H, H-10a), 1.15 (ddd, *J* = 13.2, 4.3, 2.6 Hz, 1H, H-5b), 0.99 (d, *J* = 6.8 Hz, 3H, H-14). **^{13}C NMR** (151 MHz, $CDCl_3$): δ 156.00 (C-11), 103.25 (C-13), 80.56 (C-9), 71.88 (C-1), 45.34 (C-10), 44.95 (C-12), 41.26 (C-4), 40.57 (C-3), 36.38 (C-8), 34.16 (C-2), 32.16 (C-5), 30.80 (C-6), 21.64 (C-7), 16.43 (C-14). **LR-MS** (ESI): m/z calculated for $[M+H]^+$ 223.1692, found 223.3. **Physical appearance**: white solid.

3. NMR spectra of Monicanone, Monicanol and 2-*epi*-Monicanol

3.1. *Monicanone*

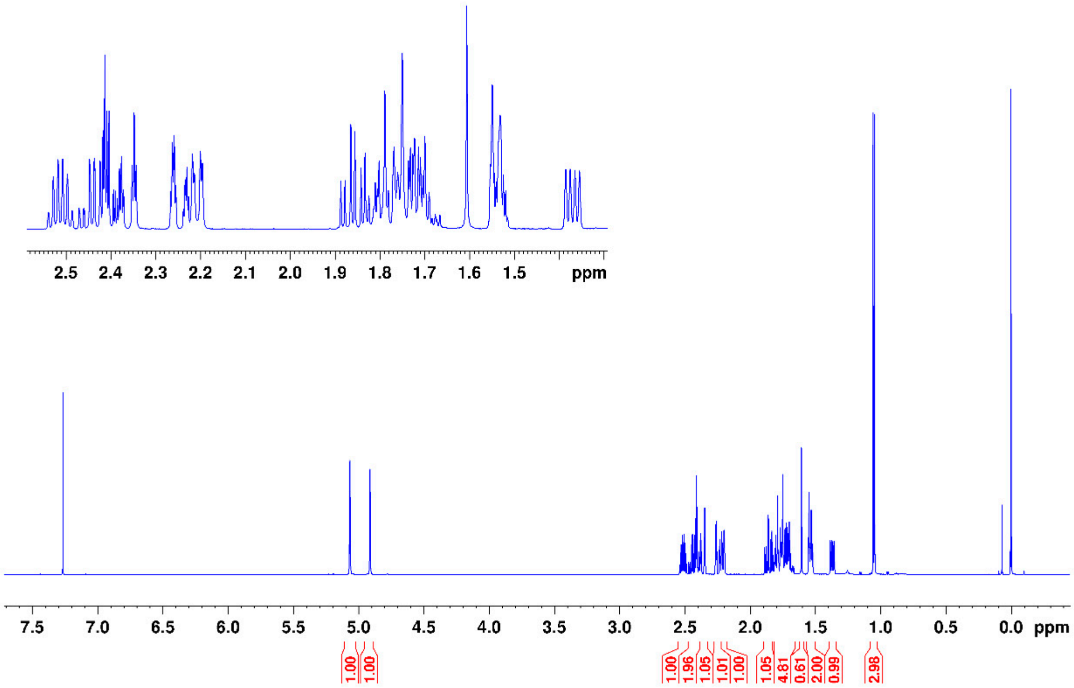

Figure S1. ^{1}H spectrum of monicanone.

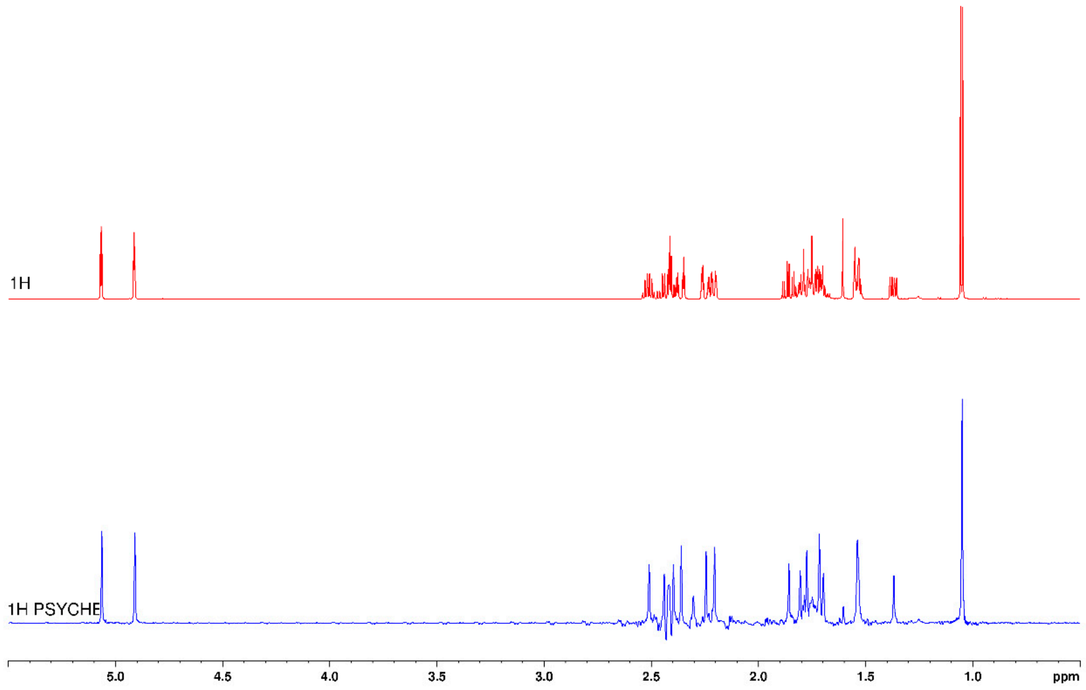

Figure S2. Stacked plot of the ^{1}H spectrum and ^{1}H PSYCHE spectrum of monicanone.

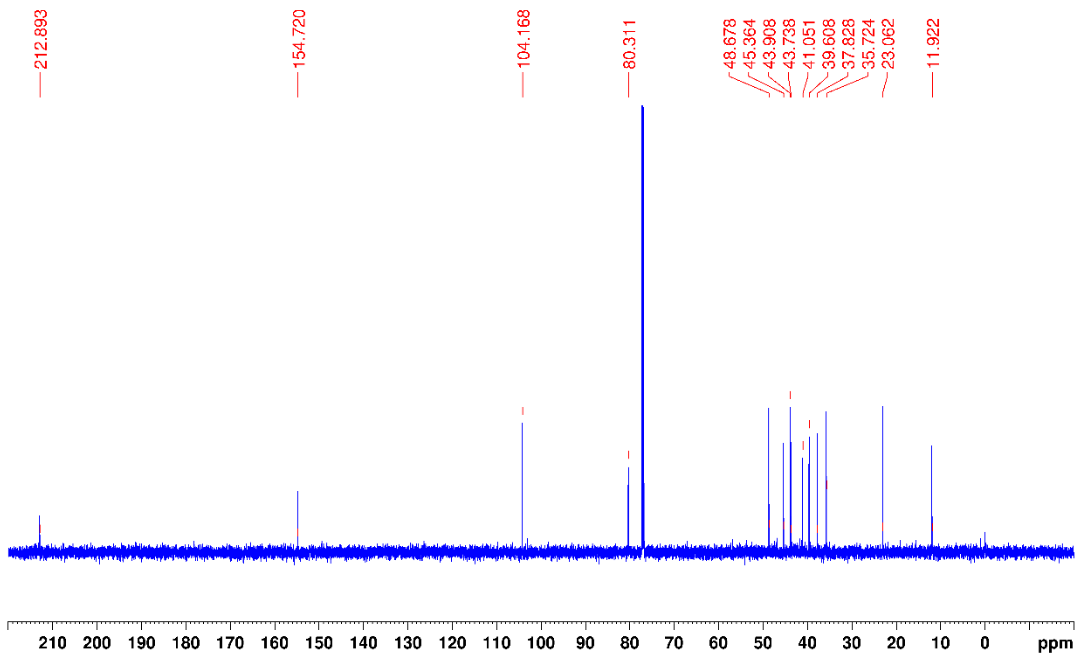

Figure S3. $^{13}C\{^{1}H\}$ spectrum of monicanone.

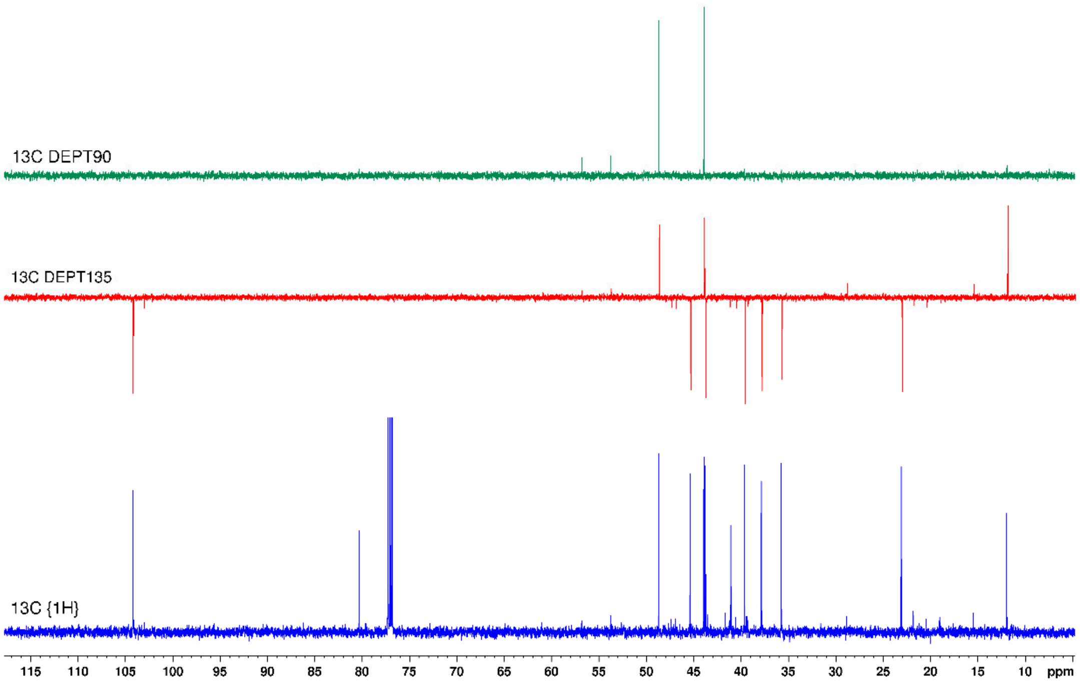

Figure S4. Stacked plot of the $^{13}C\{^{1}H\}$ spectrum, DEPT90 and DEPT135 spectrum of monicanone.

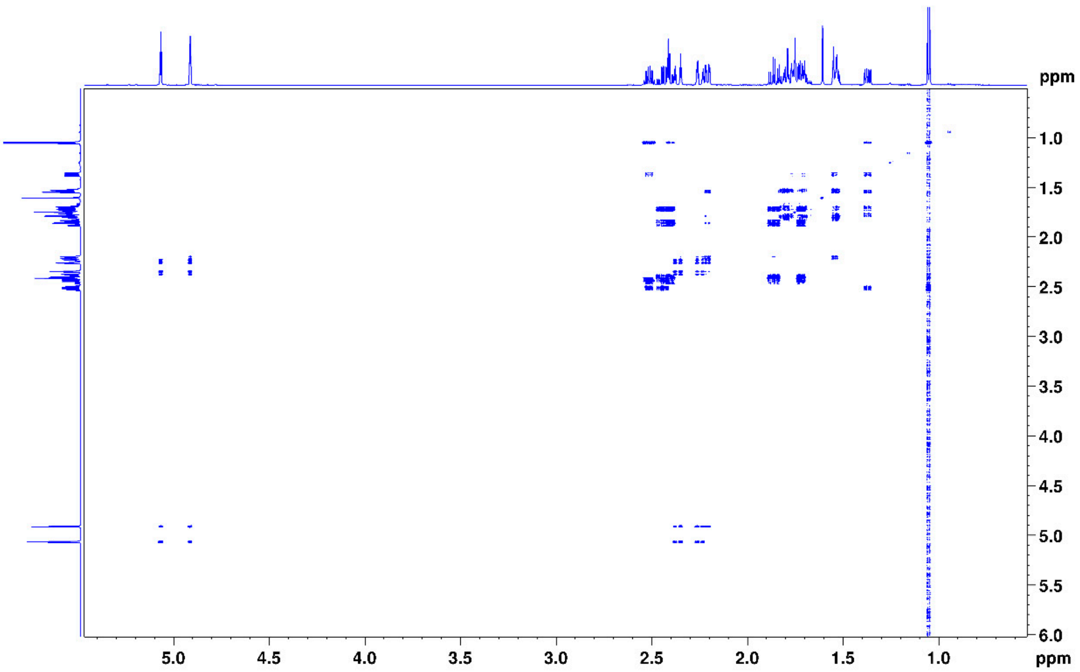

Figure S5. H-^{1}H COSY spectrum of monicanone.

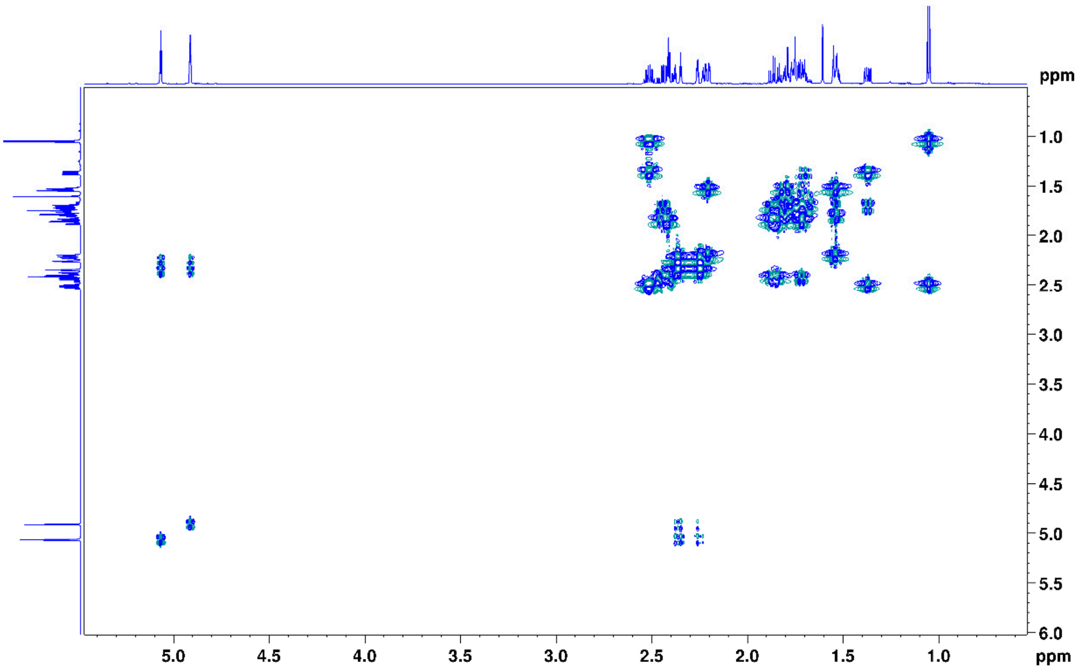

Figure S6. H-^{1}H DQF-COSY spectrum of monicanone.

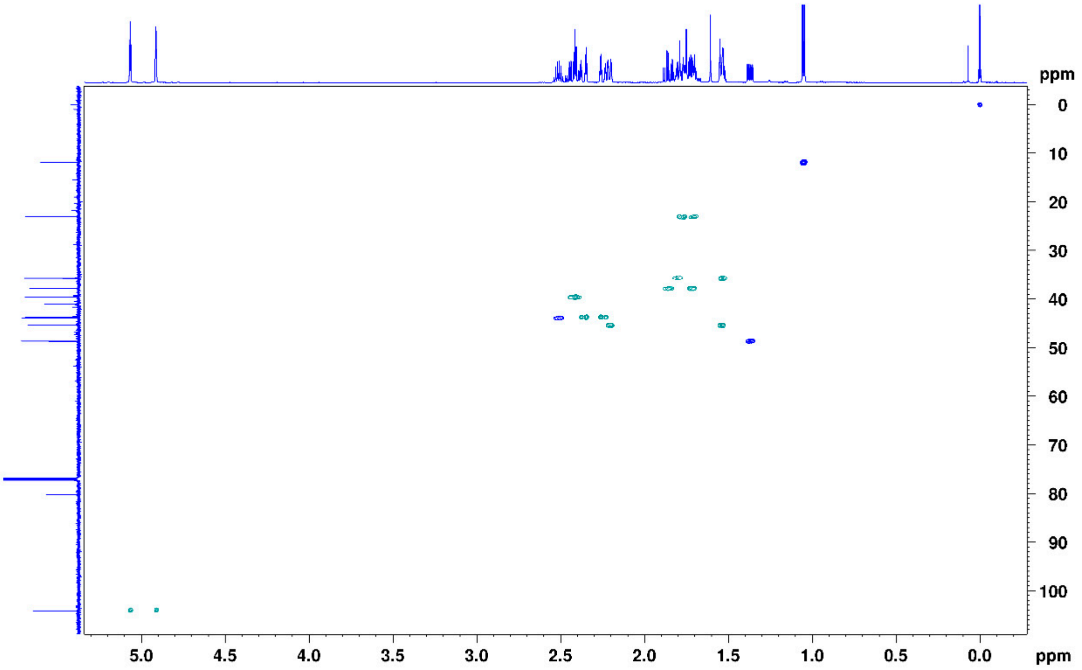

Figure S7. H-^{13}C HSQC-DEPT spectrum of monicanone.

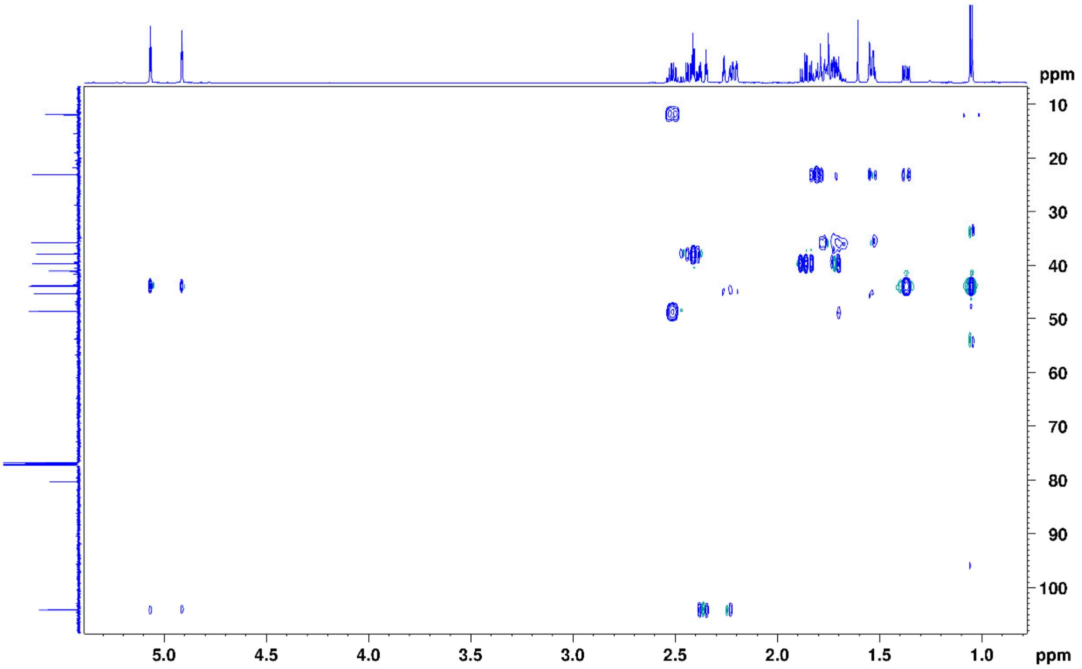

Figure S8. H-^{13}C H2BC spectrum of monicanone.

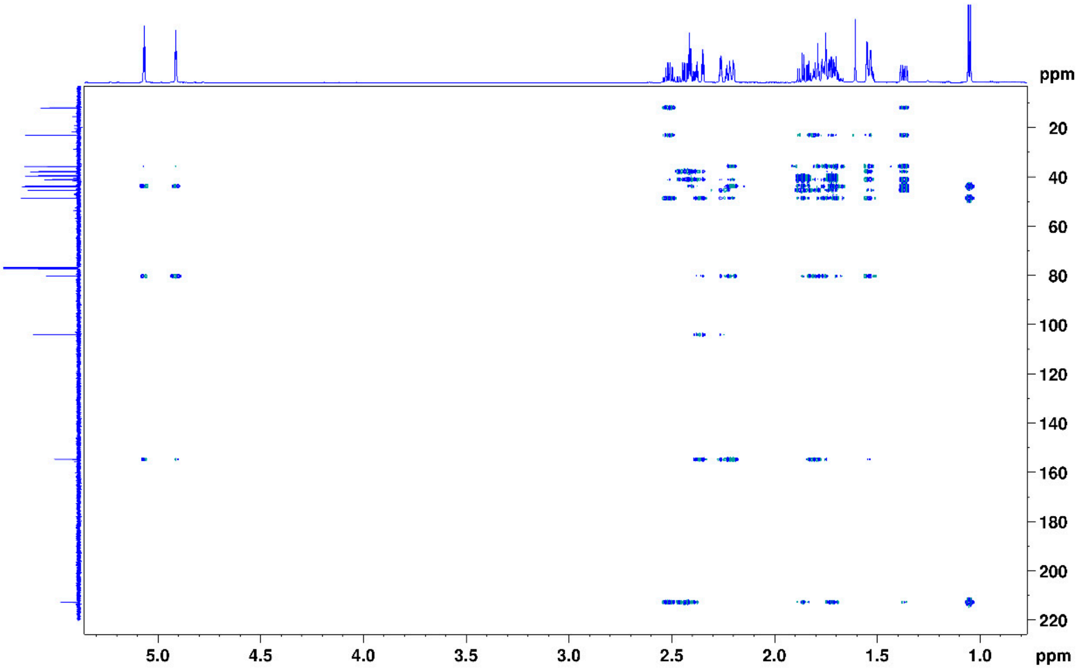

Figure S9. H-^{13}C HMBC spectrum of monicanone.

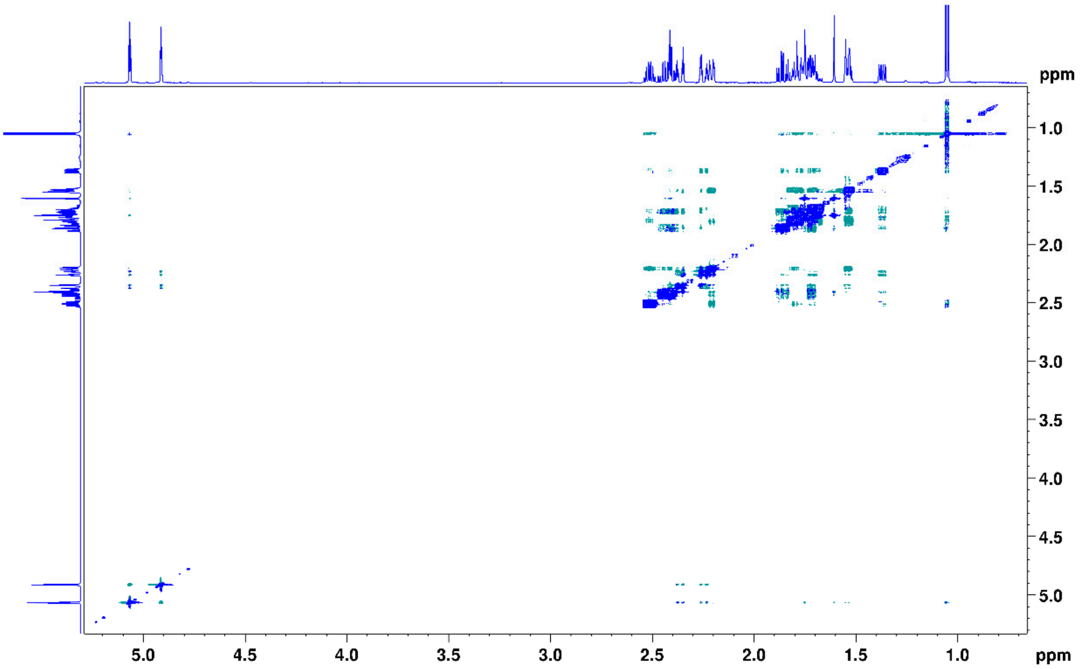

Figure S10. H-^{1}H NOESY spectrum of monicanone.

3.2. Monicanol

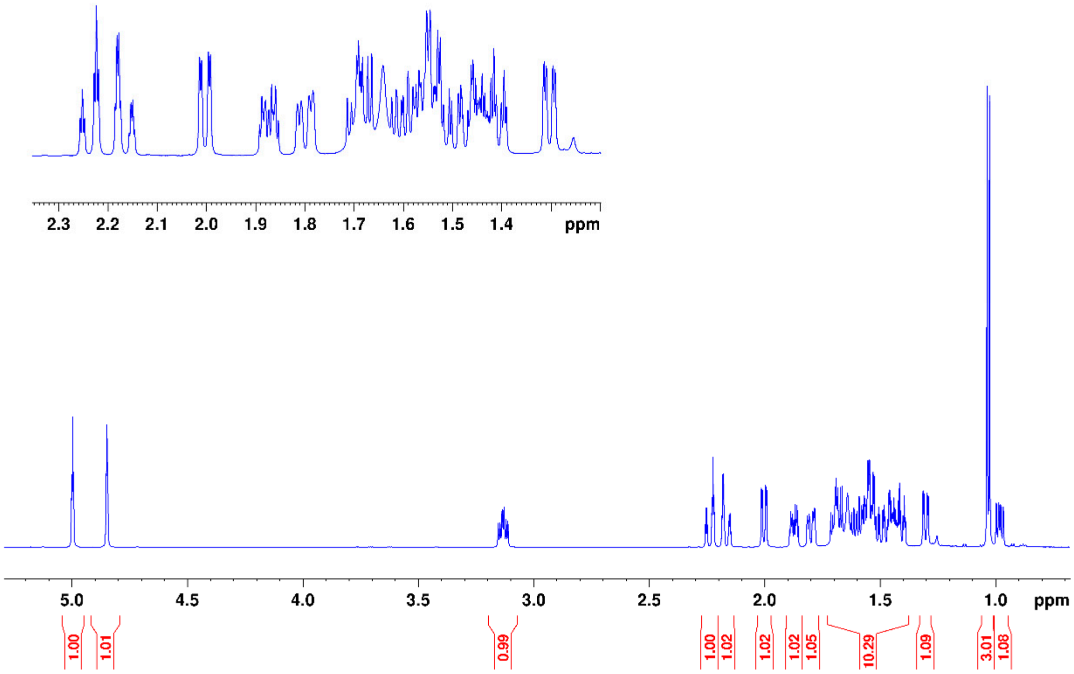

Figure S11. H spectrum of monicanol.

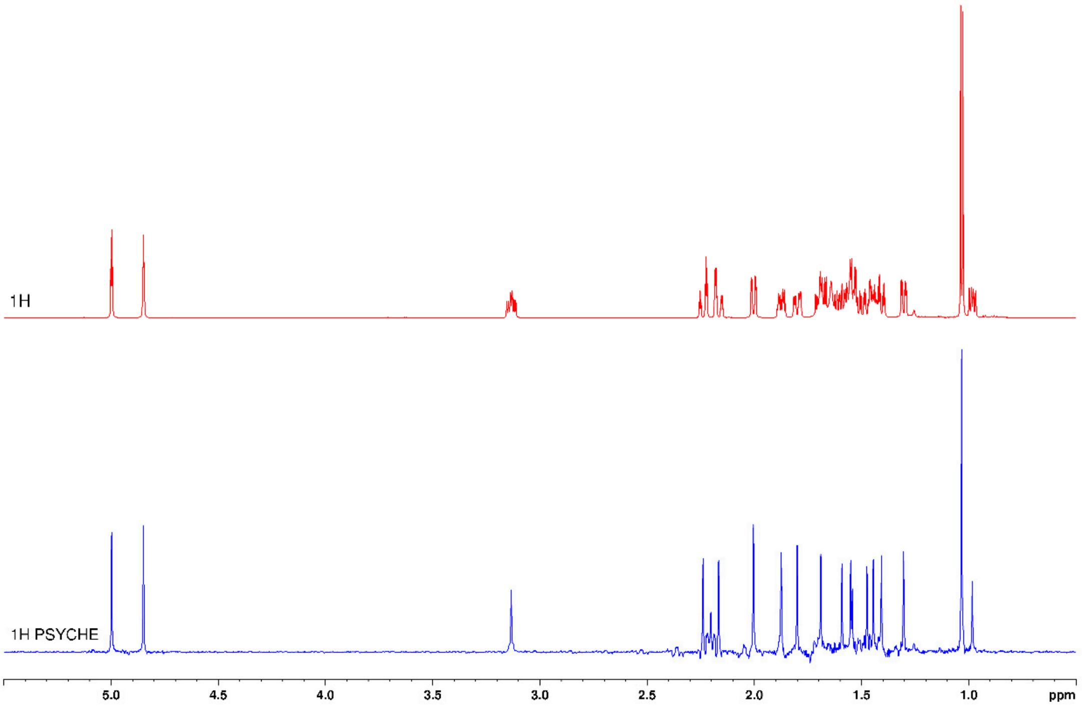

Figure S12. Stacked plot of the ^{1}H spectrum and ^{1}H PSYCHE spectrum of monicanol.

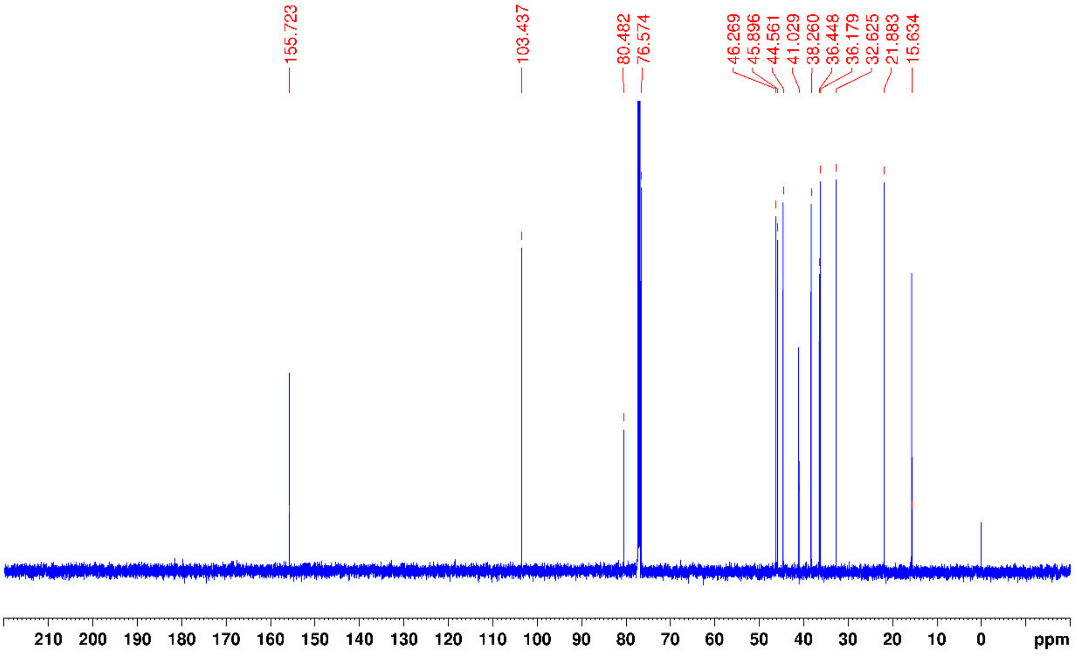

Figure S13. C{^{1}H} spectrum of monicanol.

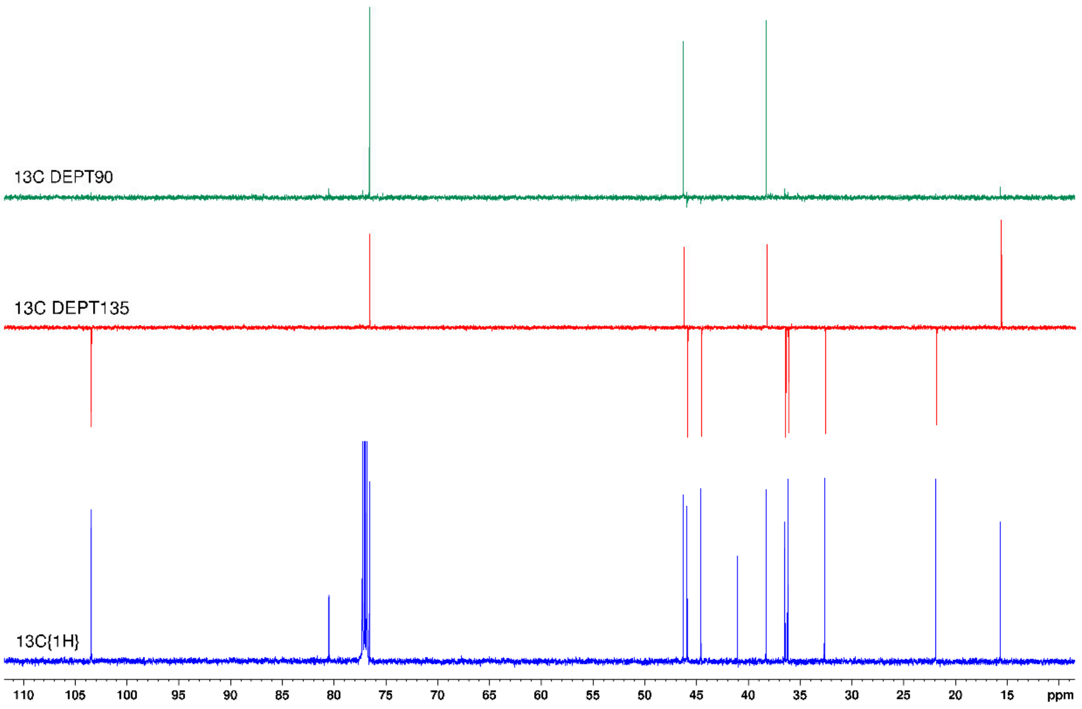

Figure S14. Stacked plot of the $^{13}C\{^{1}H\}$ spectrum, DEPT90 and DEPT135 spectrum of monicanol.

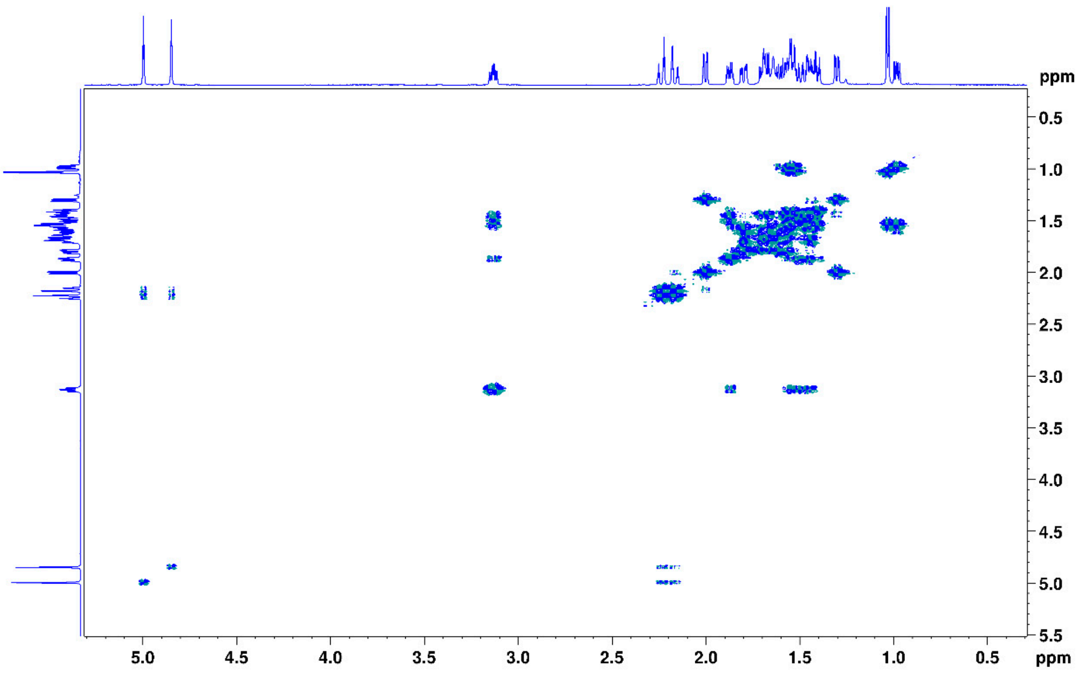

Figure S15. H-^{1}H DQF-COSY spectrum of monicanol.

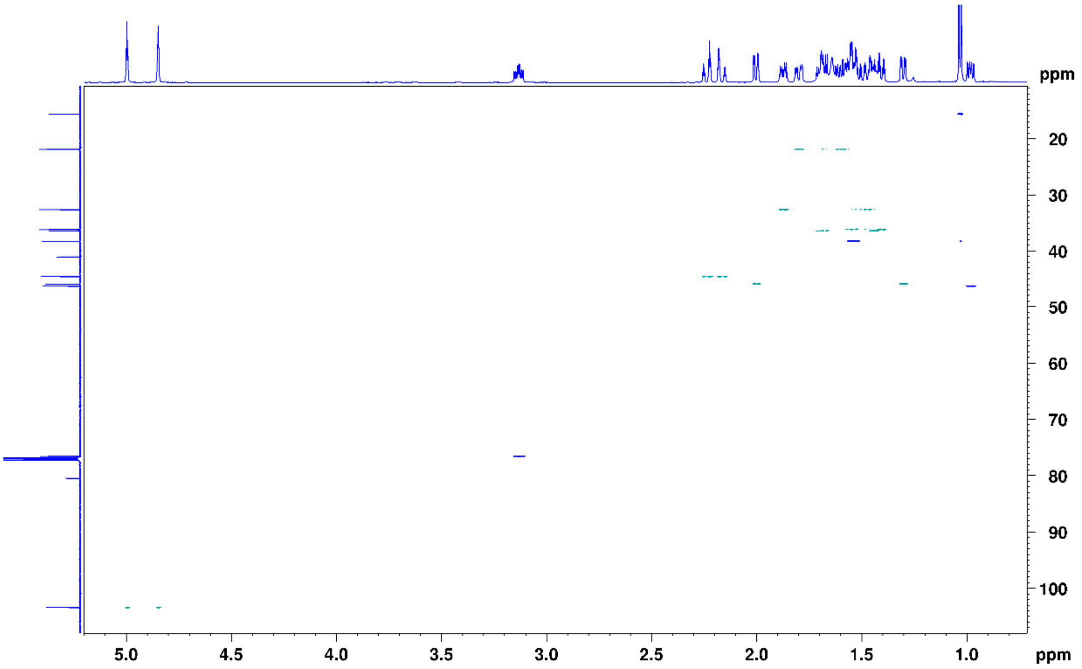

Figure S16. H-^{13}C HSQC-DEPT spectrum of monicanol.

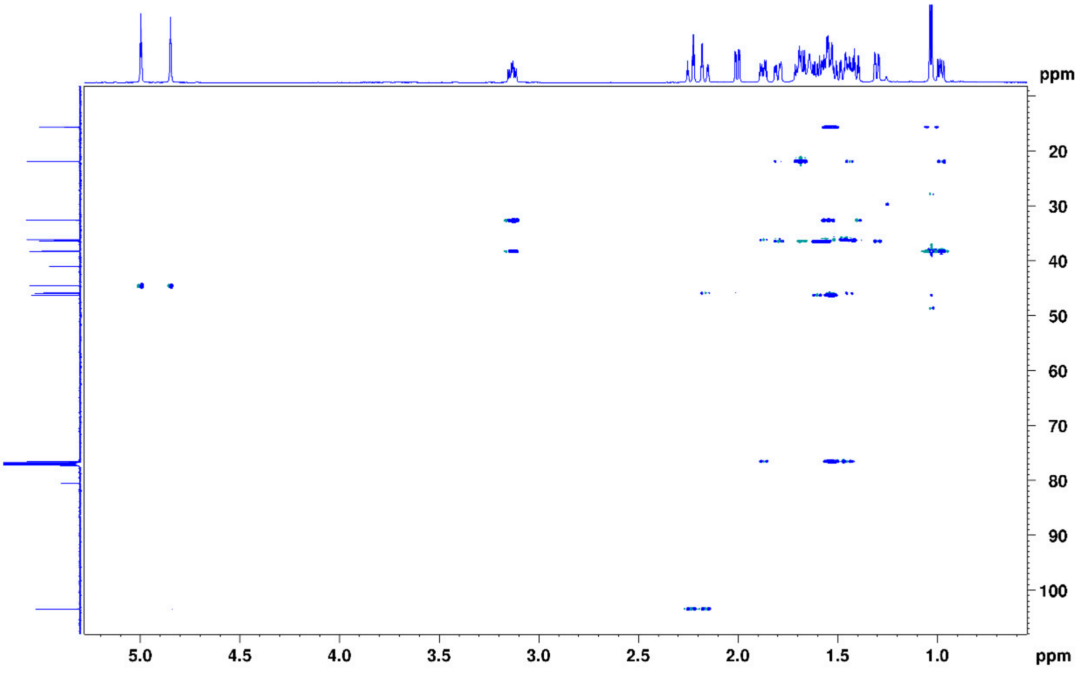

Figure S17. H-^{13}C H2BC spectrum of monicanol.

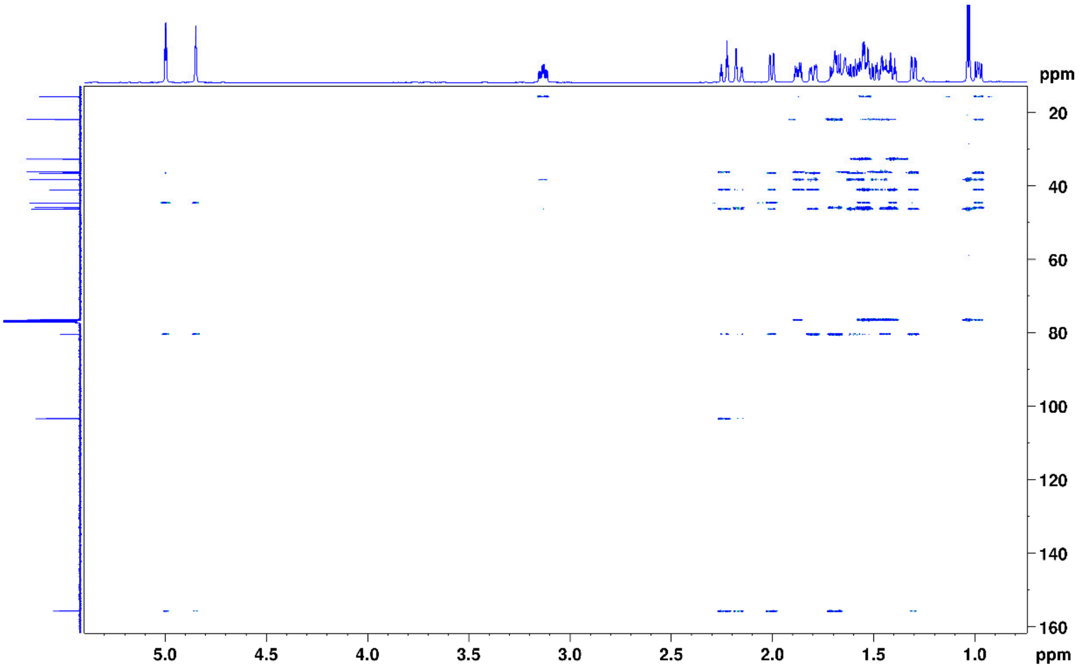

Figure S18. H-^{13}C HMBC spectrum of monicanol.

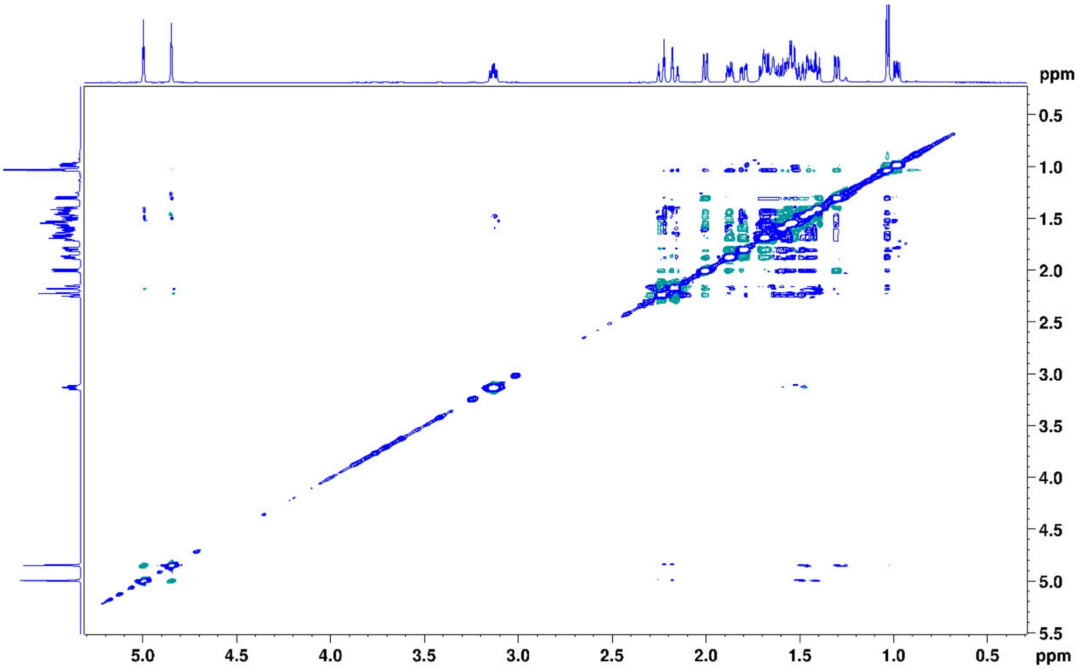

Figure S19. H-^{1}H NOESY spectrum of monicanol.

.

3.3. 2-epi-monicanol

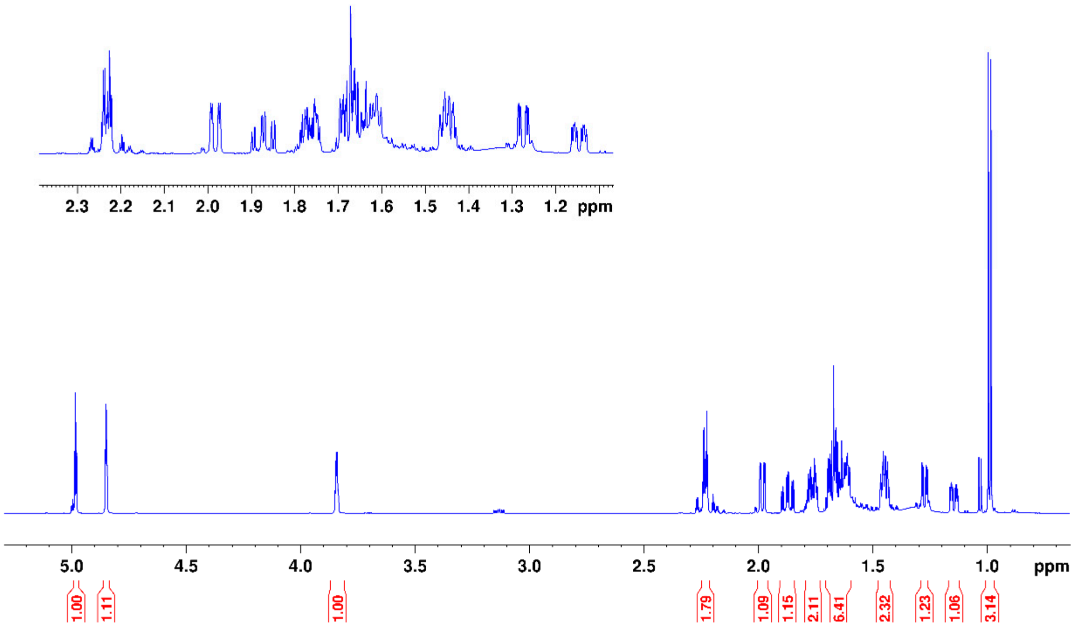

Figure S20. H spectrum of 2-*epi*-monicanol.

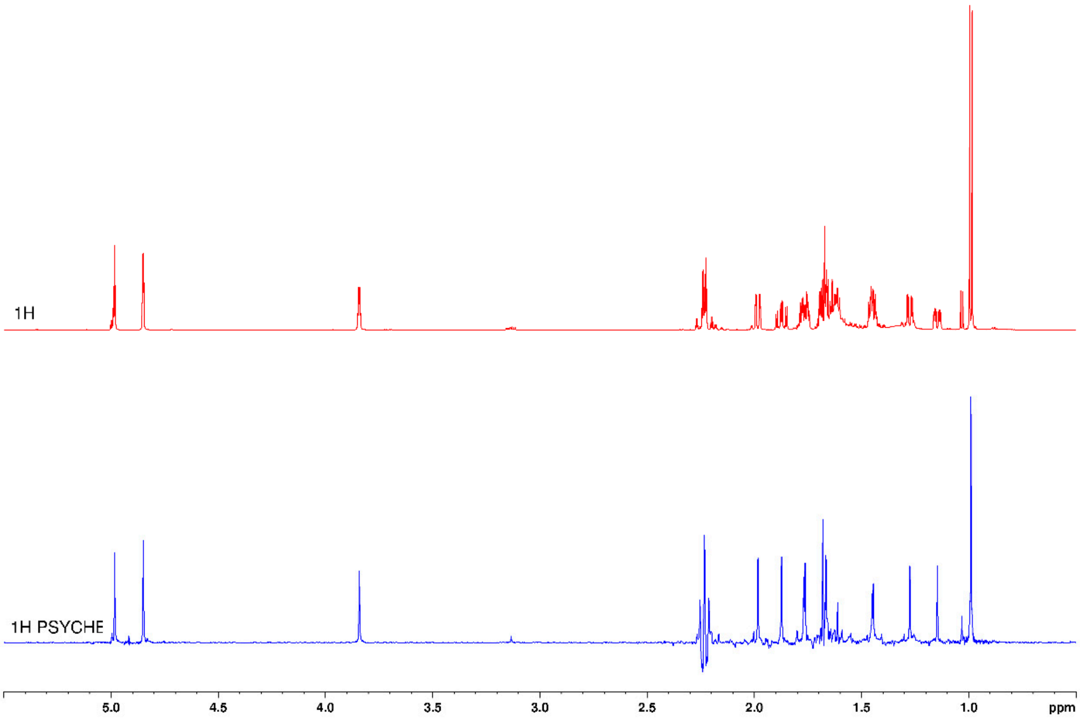

Figure S21. Stacked plot of the ^{1}H spectrum and ^{1}H PSYCHE spectrum of 2-*epi*-monicanol.

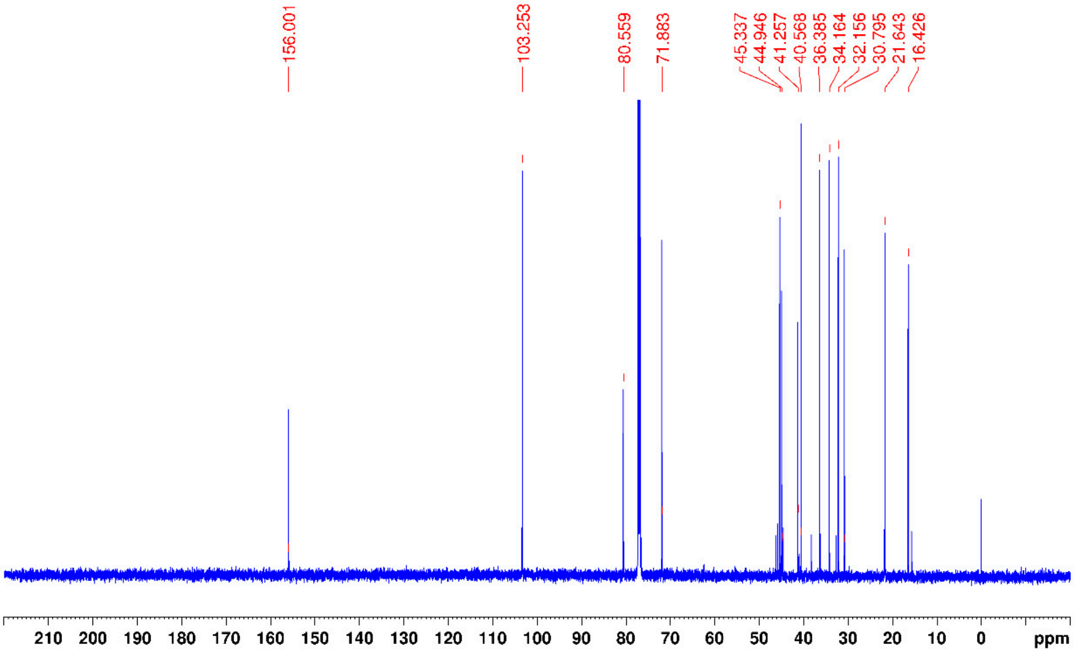

Figure S22. C{^{1}H} spectrum of 2-*epi*-monicanol.

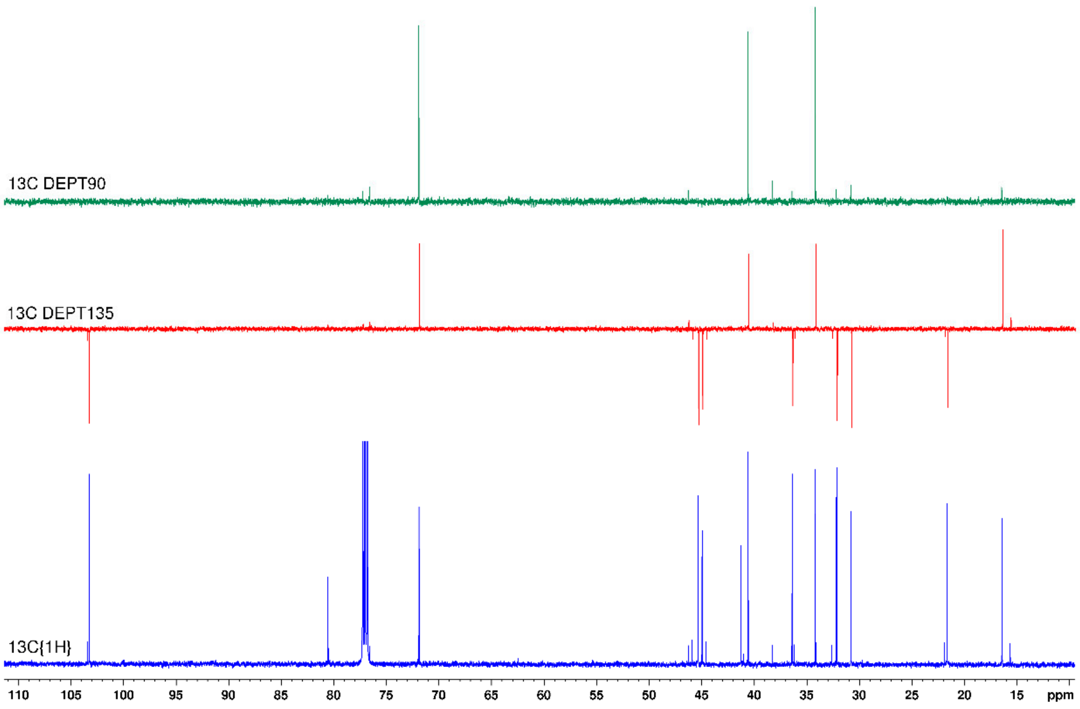

Figure S23. Stacked plot of the $^{13}C\{^{1}H\}$ spectrum, DEPT90 and DEPT135 spectrum of 2-*epi*-monicanol.

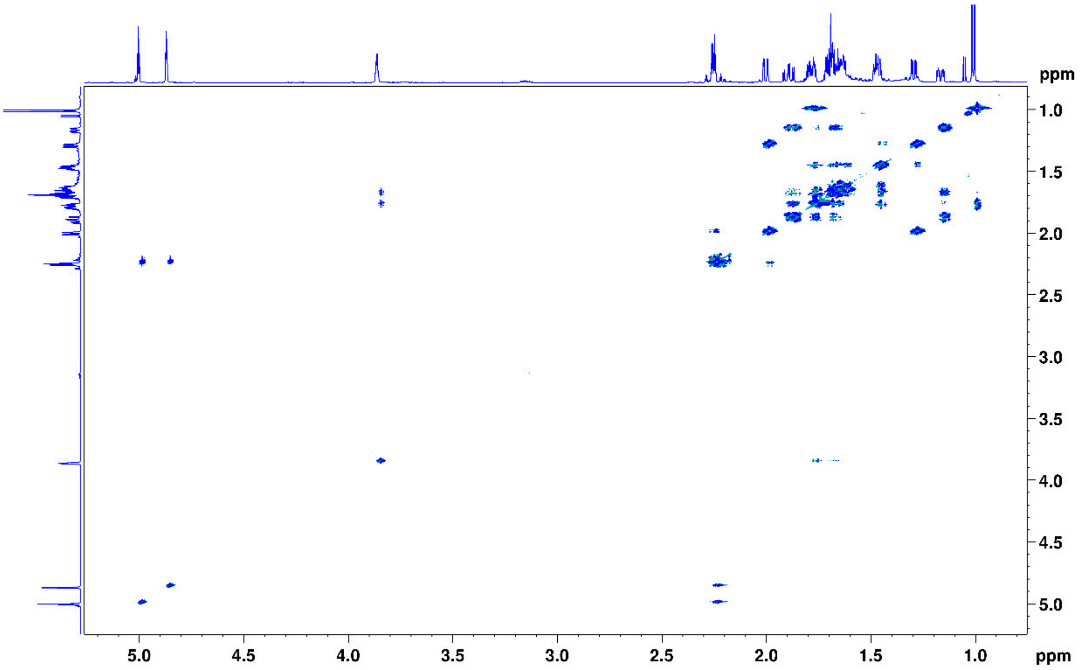

Figure S24. H-^{1}H DQF-COSY spectrum of 2-*epi*-monicanol.

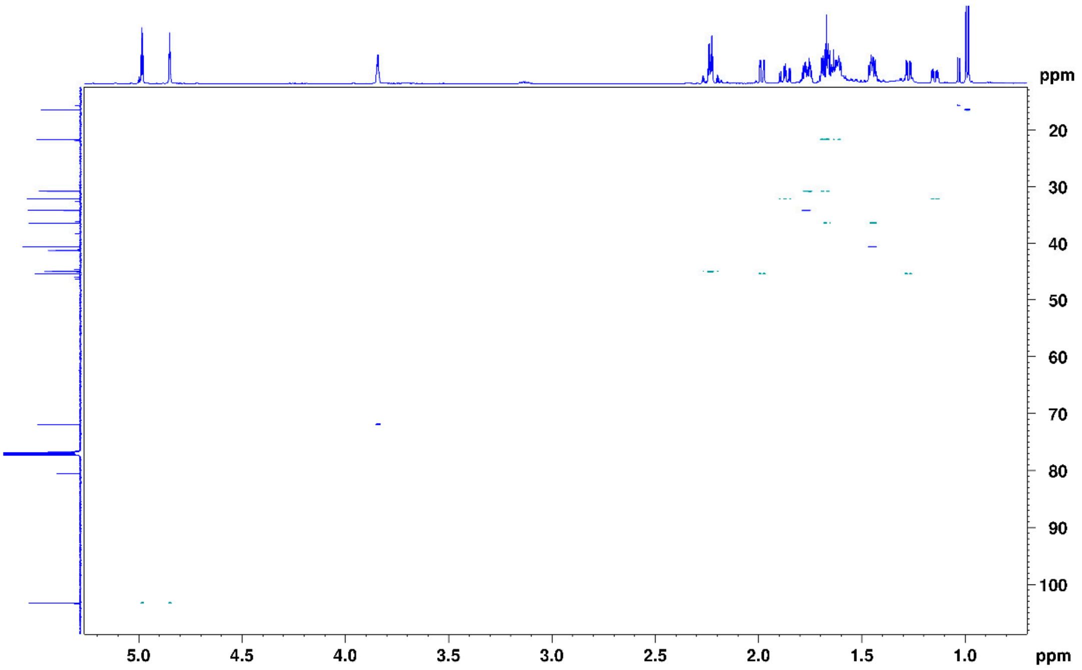

Figure S25. H-^{13}C HSQC-DEPT spectrum of 2-*epi*-monicanol.

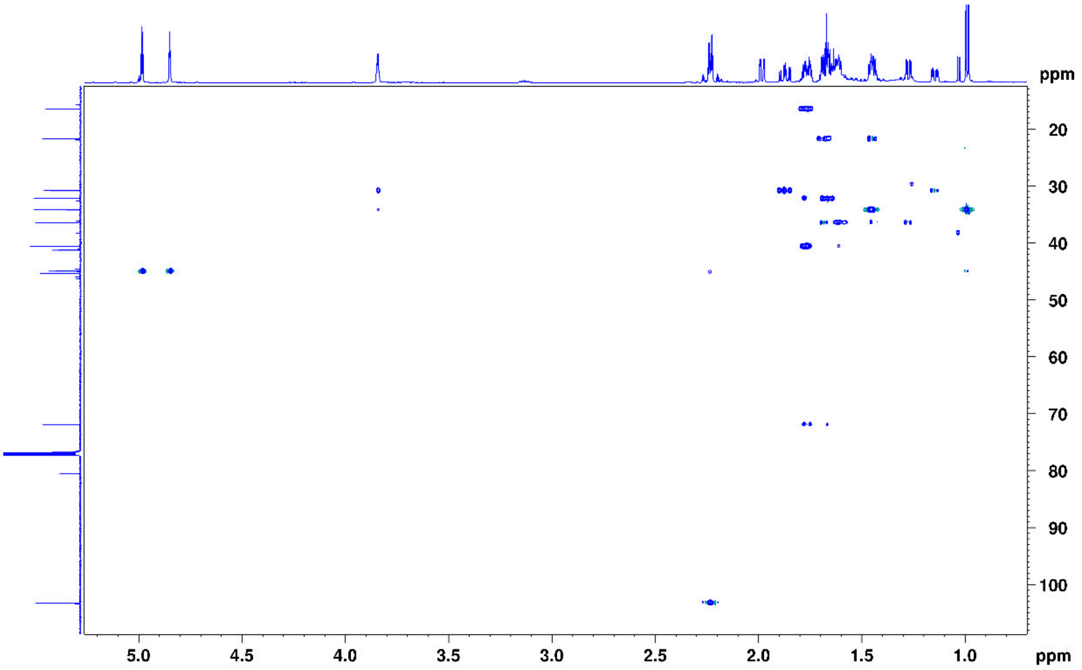

Figure S26. H-^{13}C H2BC spectrum of 2-*epi*-monicanol.

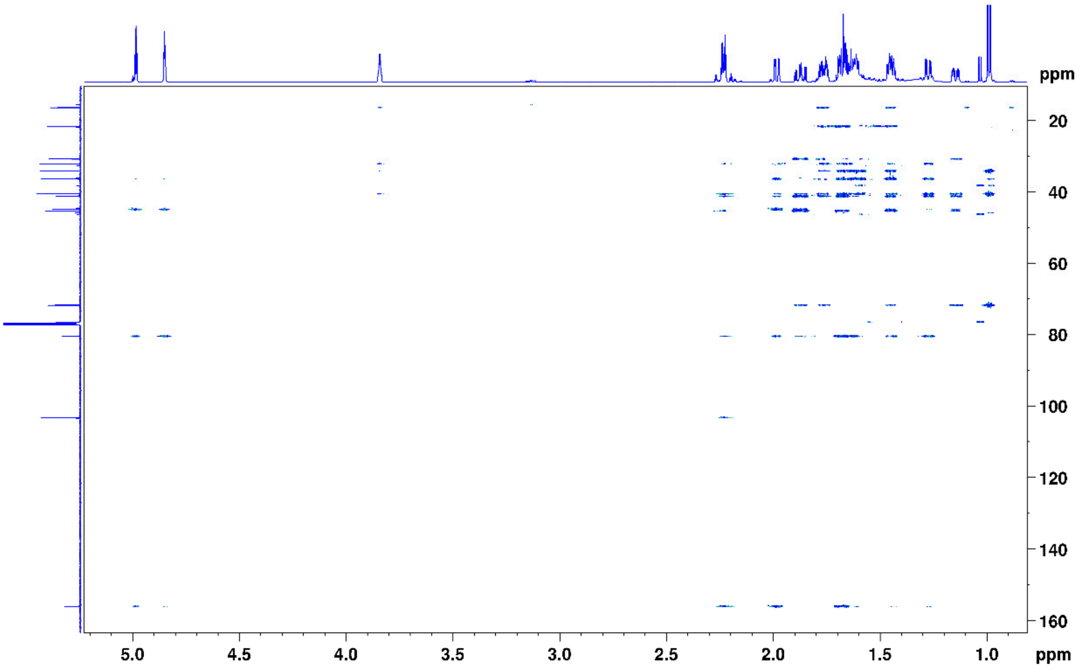

Figure S27. H-^{13}C HMBC spectrum of 2-*epi*-monicanol.

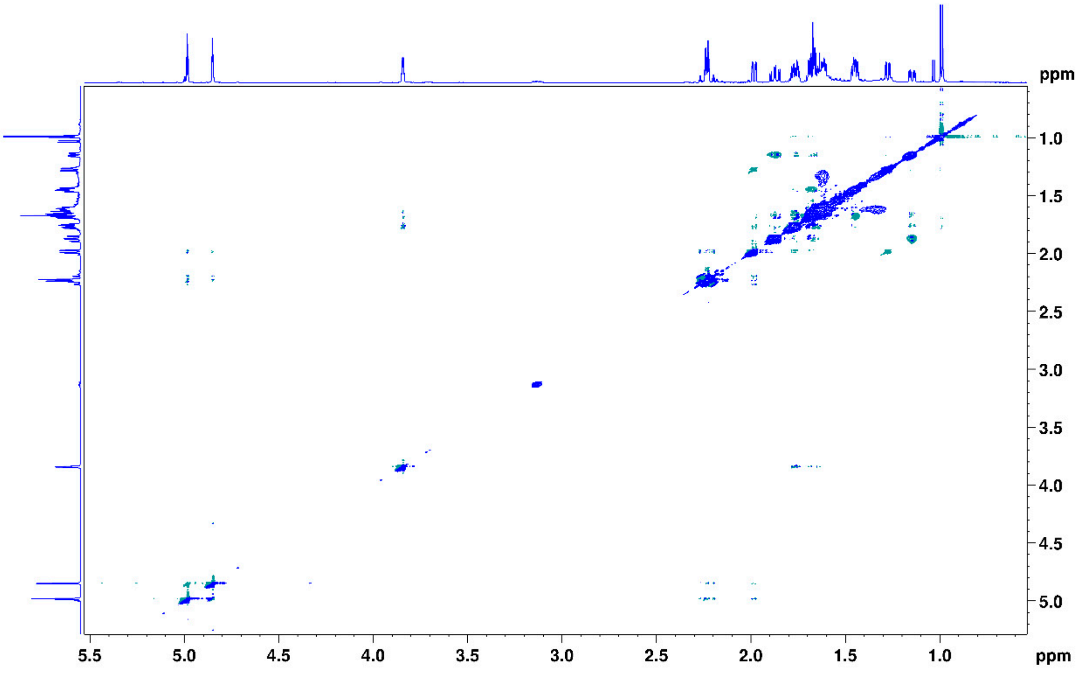

Figure S28. H-^{1}H NOESY spectrum of 2-*epi*-monicanol.

.

4. UV/Vis spectra of monicanone, monicanol and 2-*epi*-monicanol

4.1. Monicanone

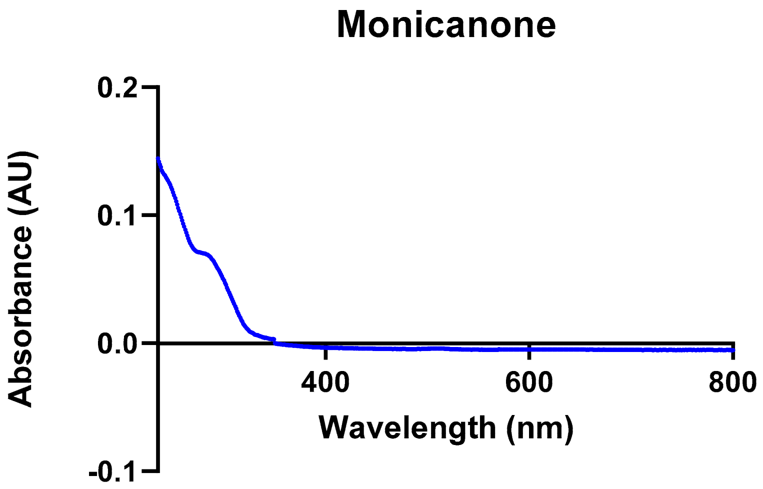

Figure S29. UV/Vis spectrum of monicanone (15.13 mM in $CDCl_3$).

4.2. Monicanol

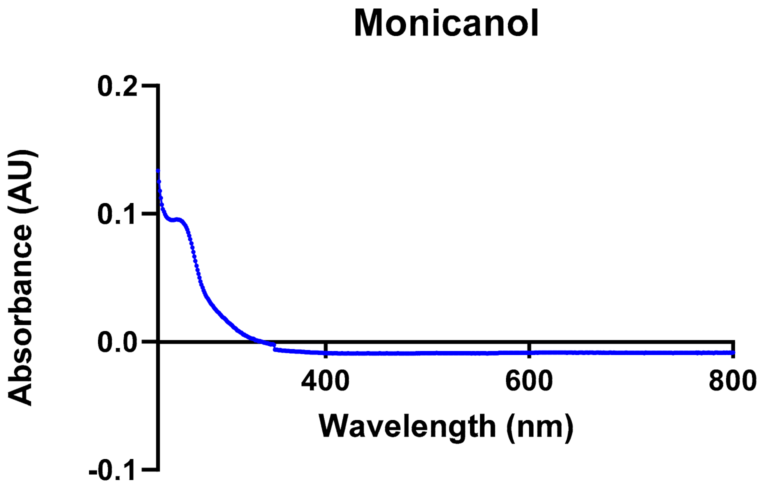

Figure S30. UV/Vis spectrum of monicanol (36.99 mM in $CDCl_3$).

4.3. 2-epi-monicanol

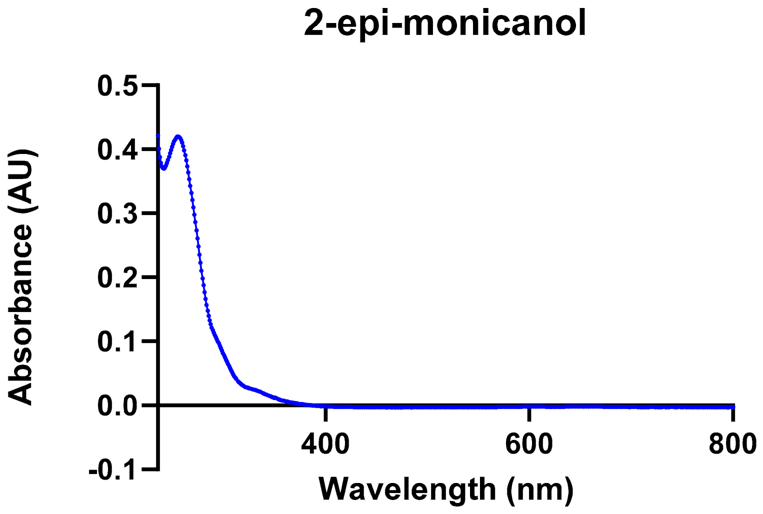

Figure S31. UV/Vis spectrum of 2-epi-monicanol (69.18 mM in $CDCl_3$).

5. CD spectra of monicanone, monicanol and 2-*epi*-monicanol

5.1. Monicanone

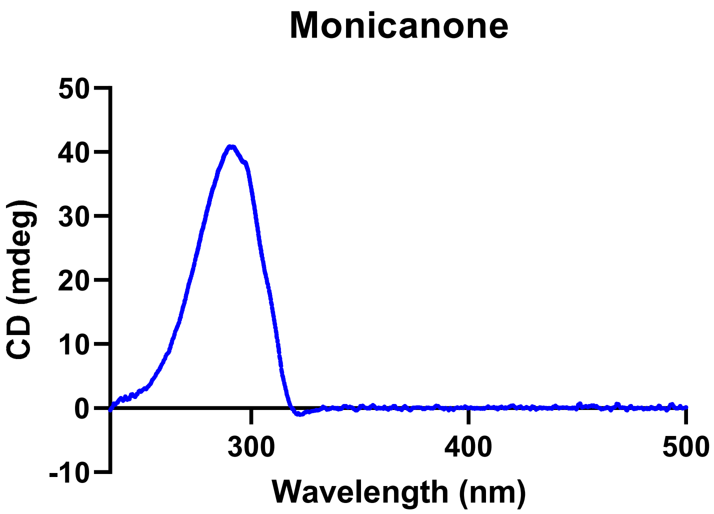

Figure S32. CD spectrum of monicanone (15.13 mM in $CDCl_3$).

5.2. Monicanol

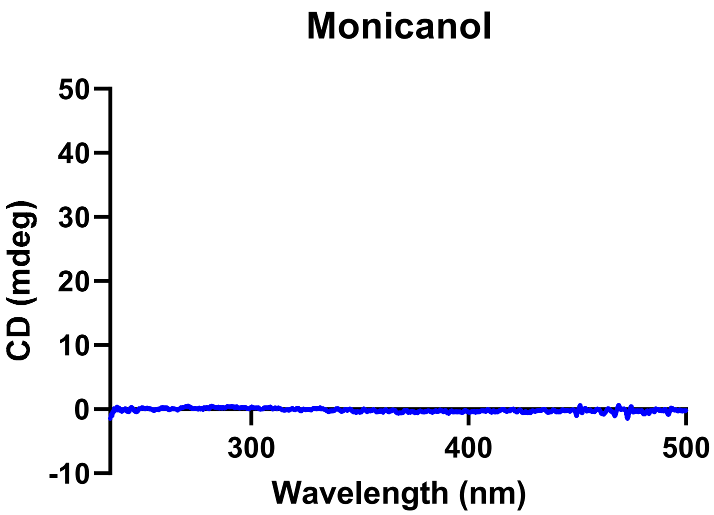

Figure S33. CD spectrum of monicanol (36.99 mM in $CDCl_3$).

5.3. 2-epi-monicanol

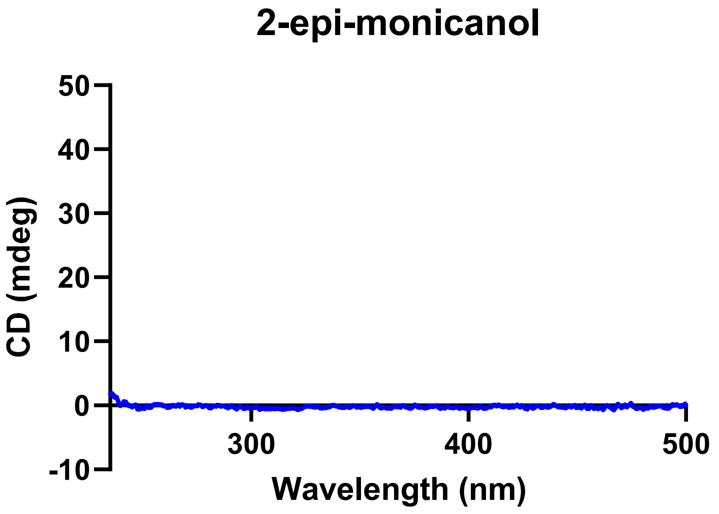

Figure S34. CD spectrum of 2-epi-monicanol (69.18 mM in $CDCl_3$).

6. XRD data of monicanone

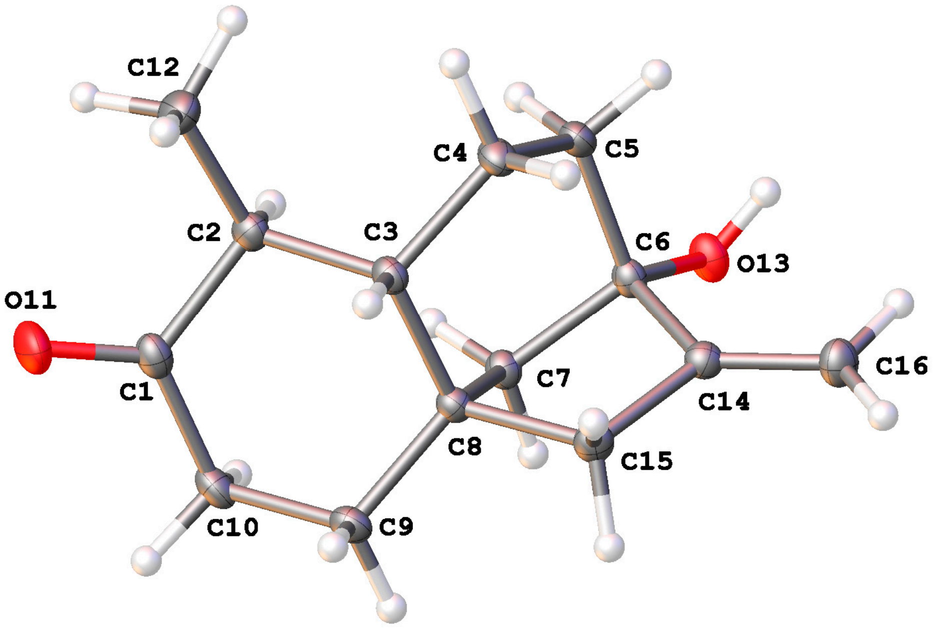

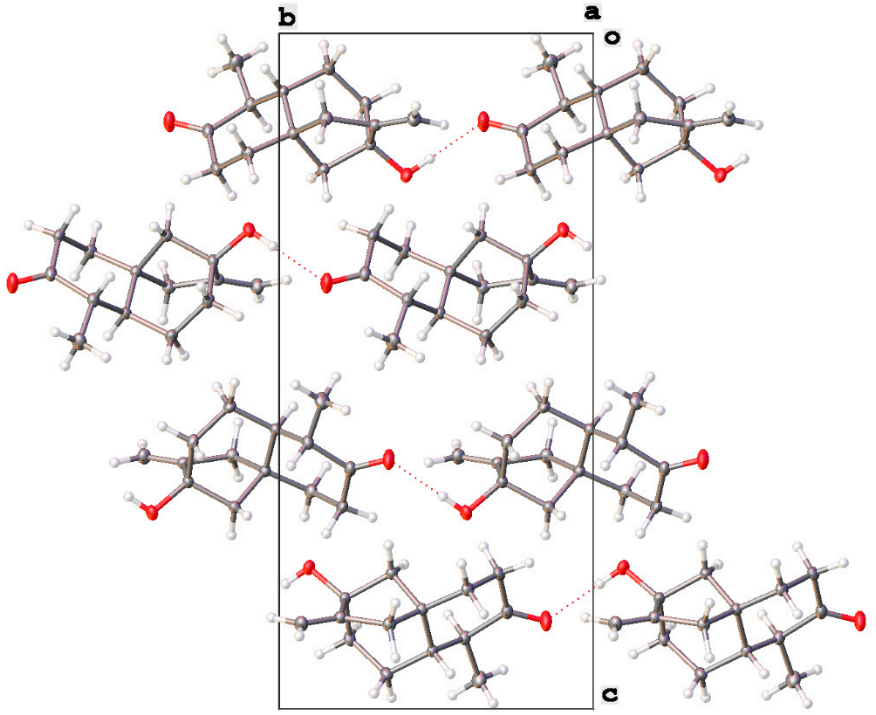

Table S1. Crystal data and structure refinement for monicanone.

Parameter	Value
Identification code	WDB1008
Empirical formula	$C_{14}H_{20}O_2$
Formula weight	220.30
Temperature (K)	294(2)
Crystal system	orthorhombic
Space group	$P2_12_12_1$
a (Å)	6.7785(3)
b (Å)	9.0461(4)
c (Å)	19.4342(10)
α (°)	90
β (°)	90
γ (°)	90
Volume ($Å^3$)	1191.69(10)
Z	4
ρ_{calc} (g/cm^3)	1.228
μ (mm^{-1})	0.080
F(000)	480.0
Crystal size (mm^3)	0.4 × 0.3 × 0.3
Radiation	Mo Kα (λ = 0.71073)
2Θ range for data collection (°)	4.966 to 52.742
Index ranges	$-8 \le h \le 8$, $-11 \le k \le 11$, $-24 \le l \le 24$
Reflections collected	12255
Independent reflections	2429 [R_{int} = 0.0232, R_{sigma} = 0.0188]
Data/restraints/parameters	2429/0/226
Goodness-of-fit on F^2	1.105
Final R indexes [I >= 2σ (I)]	R_1 = 0.0345, wR_2 = 0.0774
Final R indexes [all data]	R_1 = 0.0381, wR_2 = 0.0795
Largest diff. peak/hole (e $Å^{-3}$)	0.16/-0.12
Flack parameter	0.4(4)

Table S2. Fractional atomic coordinates (×10^4) and equivalent isotropic displacement parameters ($Å^2$×10^3) for monicanone. U_{eq} is defined as 1/3 of of the trace of the orthogonalised U_{IJ} tensor.

Atom	*x*	*y*	*z*	U(eq)
C1	3996(3)	7262(2)	3556.0(12)	41.4(5)
C2	4741(3)	5913(2)	3946.2(12)	37.7(5)
C3	3002(3)	4882(2)	4140.3(11)	33.3(5)
C4	3667(4)	3425(2)	4477.9(12)	40.7(5)
C5	4284(3)	2248(2)	3956.2(12)	39.6(5)
C6	2806(3)	2102(2)	3365.1(11)	32.9(5)
C7	2698(3)	3583(2)	2991.0(11)	33.4(5)
C8	1627(3)	4554(2)	3523.9(10)	31.8(4)
C9	852(4)	6005(2)	3227.1(13)	39.6(5)

Atom	*x*	*y*	*z*	U(eq)
C10	2535(4)	7001(2)	2993.6(12)	42.9(6)
O11	4590(3)	8490.1(19)	3695.9(11)	68.5(6)
C12	5995(5)	6367(3)	4562.0(16)	56.3(7)
O13	3372(3)	951.1(18)	2906.6(8)	44.0(4)
C14	719(3)	1953(2)	3642.4(10)	34.6(5)
C15	-68(3)	3509(2)	3748.0(12)	38.6(5)
C16	-227(4)	702(3)	3738.9(13)	45.6(6)

Table S3. Anisotropic displacement parameters ($Å^2×10^3$) for monicanone. The anisotropic displacement factor exponent takes the form: $-2\pi^2[h^2a^{*2}U_{11}+2hka^*b^*U_{12}+…]$.

Atom	U_{11}	U_{22}	U_{33}	U_{23}	U_{13}	U_{12}
C1	45.3(13)	29.6(10)	49.2(13)	0.5(9)	7.9(11)	2.6(10)
C2	37.5(11)	30.5(10)	45.0(12)	-2.3(9)	-1.6(10)	2.5(9)
C3	39.4(12)	28.2(10)	32.3(11)	-0.7(8)	0.2(9)	3.1(9)
C4	50.4(13)	35.2(11)	36.6(11)	5.5(9)	-8.8(11)	-0.1(11)
C5	37.2(12)	30.2(11)	51.4(13)	5.5(10)	-6.0(11)	4.1(10)
C6	35.4(11)	26.7(9)	36.5(10)	-0.9(9)	4.1(9)	1.8(9)
C7	37.7(11)	30.8(10)	31.8(11)	-0.1(8)	2.4(9)	0.3(9)
C8	32.4(10)	30.1(10)	32.9(10)	1.5(8)	0.2(8)	2.2(9)
C9	40.5(12)	35.1(11)	43.3(12)	1.8(10)	-2.3(11)	9.6(9)
C10	56.4(15)	30.2(11)	42.2(12)	7.4(10)	1.2(11)	7.4(11)
O11	85.5(15)	29.2(9)	90.7(14)	2.0(9)	-18.0(12)	-5.2(10)
C12	56.5(17)	47.0(14)	65.5(18)	-4.3(13)	-17.2(15)	-6.4(14)
O13	52.3(9)	30.9(8)	48.8(10)	-5.4(7)	10.4(8)	4.2(7)
C14	35.8(10)	35.7(11)	32.2(10)	1.6(9)	0.5(9)	0.3(9)
C15	32.5(10)	38.5(11)	44.7(13)	-0.4(10)	5.1(10)	2.1(10)
C16	44.7(13)	40.5(13)	51.5(14)	0.2(11)	9.4(11)	-5.4(11)

Table S4. Bond lengths for monicanone.

Atom	Atom	Length (Å)	Atom	Atom	Length (Å)
C1	C2	1.523(3)	C6	C7	1.526(3)
C1	C10	1.494(3)	C6	O13	1.423(2)
C1	O11	1.213(3)	C6	C14	1.520(3)
C2	C3	1.550(3)	C7	C8	1.540(3)
C2	C12	1.525(3)	C8	C9	1.526(3)
C3	C4	1.539(3)	C8	C15	1.550(3)
C3	C8	1.547(3)	C9	C10	1.523(3)
C4	C5	1.528(3)	C14	C15	1.520(3)
C5	C6	1.530(3)	C14	C16	1.314(3)

Table S5. Bond angles for monicanone**.**

Atom	Atom	Atom	Angle (˚)	Atom	Atom	Atom	Angle (˚)
C10	C1	C2	117.25(18)	C14	C6	C5	110.54(17)
O11	C1	C2	120.8(2)	C14	C6	C7	101.66(16)
O11	C1	C10	121.9(2)	C6	C7	C8	101.73(16)
C1	C2	C3	110.58(18)	C3	C8	C15	110.24(17)
C1	C2	C12	111.1(2)	C7	C8	C3	110.23(17)
C12	C2	C3	113.3(2)	C7	C8	C15	100.97(17)
C4	C3	C2	113.35(18)	C9	C8	C3	109.59(17)
C4	C3	C8	110.04(16)	C9	C8	C7	113.49(17)
C8	C3	C2	112.66(17)	C9	C8	C15	112.06(18)
C5	C4	C3	113.18(18)	C10	C9	C8	111.34(18)
C4	C5	C6	112.29(18)	C1	C10	C9	111.83(19)
C7	C6	C5	108.25(17)	C15	C14	C6	106.99(17)
O13	C6	C5	110.91(17)	C16	C14	C6	125.5(2)
O13	C6	C7	110.91(17)	C16	C14	C15	127.4(2)
O13	C6	C14	114.09(17)	C14	C15	C8	105.50(17)

Table S6. Torsion angles for monicanone.

A	B	C	D	Angle (˚)	A	B	C	D	Angle (˚)
C1	C2	C3	C4	174.72(18)	C6	C14	C15	C8	0.0(2)
C1	C2	C3	C8	48.9(2)	C7	C6	C14	C15	-28.2(2)
C2	C1	C10	C9	48.7(3)	C7	C6	C14	C16	149.0(2)
C2	C3	C4	C5	-82.8(2)	C7	C8	C9	C10	-65.0(2)
C2	C3	C8	C7	68.9(2)	C7	C8	C15	C14	27.9(2)
C2	C3	C8	C9	-56.7(2)	C8	C3	C4	C5	44.4(3)
C2	C3	C8	C15	179.50(17)	C8	C9	C10	C1	-54.3(3)
C3	C4	C5	C6	-46.4(3)	C9	C8	C15	C14	149.05(18)
C3	C8	C9	C10	58.7(2)	C10	C1	C2	C3	-45.6(3)
C3	C8	C15	C14	-88.6(2)	C10	C1	C2	C12	-172.3(2)
C4	C3	C8	C7	-58.7(2)	O11	C1	C2	C3	135.1(2)
C4	C3	C8	C9	175.74(19)	O11	C1	C2	C12	8.4(3)
C4	C3	C8	C15	51.9(2)	O11	C1	C10	C9	-132.0(2)
C4	C5	C6	C7	60.9(2)	C12	C2	C3	C4	-59.8(3)
C4	C5	C6	O13	-177.22(18)	C12	C2	C3	C8	174.40(19)
C4	C5	C6	C14	-49.6(2)	O13	C6	C7	C8	167.53(16)
C5	C6	C7	C8	-70.6(2)	O13	C6	C14	C15	-147.67(19)
C5	C6	C14	C15	86.5(2)	O13	C6	C14	C16	29.6(3)
C5	C6	C14	C16	-96.3(3)	C14	C6	C7	C8	45.86(19)
C6	C7	C8	C3	70.9(2)	C15	C8	C9	C10	-178.60(19)
C6	C7	C8	C9	-165.71(17)	C16	C14	C15	C8	-177.2(2)
C6	C7	C8	C15	-45.6(2)					

Table S7. Hydrogen atom coordinates (Å×10[4]) and isotropic displacement parameters (Å[2]×10[3]) for monicanone.

Atom	*x*	*y*	*z*	U(eq)
H16A	-1550(40)	640(30)	3921(14)	59(7)
H16B	480(40)	-240(30)	3647(12)	51(7)
H13	3680(40)	210(30)	3146(14)	55(8)
H3	2170(30)	5440(20)	4477(12)	39(6)
H2	5560(30)	5380(20)	3602(10)	30(5)
H10A	2050(40)	7960(30)	2836(12)	44(6)
H10B	3210(40)	6500(30)	2599(13)	49(7)
H9A	50(40)	6510(20)	3596(12)	39(6)
H9B	-50(40)	5810(30)	2848(12)	43(6)
H7A	4010(40)	3970(30)	2861(12)	43(6)
H7B	1900(40)	3480(30)	2576(12)	44(6)
H15A	-1250(40)	3710(20)	3432(12)	41(6)
H15B	-530(40)	3700(30)	4241(13)	46(7)
H5A	5570(40)	2480(20)	3745(11)	36(6)
H5B	4470(40)	1280(30)	4187(12)	42(6)
H4A	2570(40)	3020(30)	4768(13)	51(7)
H4B	4780(40)	3580(30)	4830(12)	46(7)
H12A	6720(60)	5520(40)	4798(18)	90(10)
H12B	5240(50)	6810(30)	4917(16)	79(10)
H12C	6970(60)	7130(40)	4420(18)	99(12)
